# Defect evolution and electrical response in HAp–TiO_2_-derived calcium phosphate–titanate composites investigated by positron annihilation lifetime spectroscopy

**DOI:** 10.1039/d6ra04627d

**Published:** 2026-08-24

**Authors:** Uğur Yahşi, Şeyma Şimal Ökmen, Fatih Dumludağ, Cumali Tav, Ziya Engin Erkmen

**Affiliations:** a Physics Department, Faculty of Science, Marmara University 34722 Kadikoy Istanbul Türkiye uyahsi@marmara.edu.tr; b Metallurgical and Materials Engineering Department, Faculty of Engineering, Marmara University 34854 Maltepe Istanbul Türkiye zeerkmen@gmail.com

## Abstract

The structural evolution of hydroxyapatite (HAp)–TiO_2_ composites was investigated as a function of TiO_2_ content (0–5 wt%) and sintering temperature (1000–1300 °C). X-ray diffraction revealed that TiO_2_ no longer exists as a separate phase above ∼1100 °C owing to solid-state reactions with HAp, resulting in the formation of β-tricalcium phosphate (β-TCP) and calcium titanate phases (CaTiO_3_/Ca_4_Ti_3_O_10_). Consequently, the resulting materials consist of multiphase calcium phosphate–titanate composites rather than TiO_2_-doped HAp. SEM observations showed significant temperature-dependent microstructural evolution, including densification, grain growth, and the formation of interfacial regions between the constituent phases. Positron annihilation lifetime spectroscopy (PALS) revealed systematic variations in the defect-related lifetime (*τ*_2_ = 0.44–0.62 ns) and intensity, suggesting an evolution from pore-dominated defects to increasingly localized interfacial trapping sites during phase transformation. Electrical measurements showed low bulk conductivity, with the non-monotonic DC conductivity behavior reflecting the combined influence of defect evolution, interfacial heterogeneity, and microstructural changes, while the AC conductivity response suggested localized hopping and polarization processes. The combined use of XRD, SEM, PALS, and electrical measurements provides new insight into the interplay among phase evolution, defect structure, microstructure, and electrical behavior in HAp–TiO_2_-derived calcium phosphate–titanate composites.

## Abstract 2

Combined XRD, SEM, PALS, and electrical analyses reveal phase and defect evolution in HAp–TiO_2_-derived calcium phosphate–titanate ceramics and their relationship with microstructure and electrical behavior.

## Graphical abstract

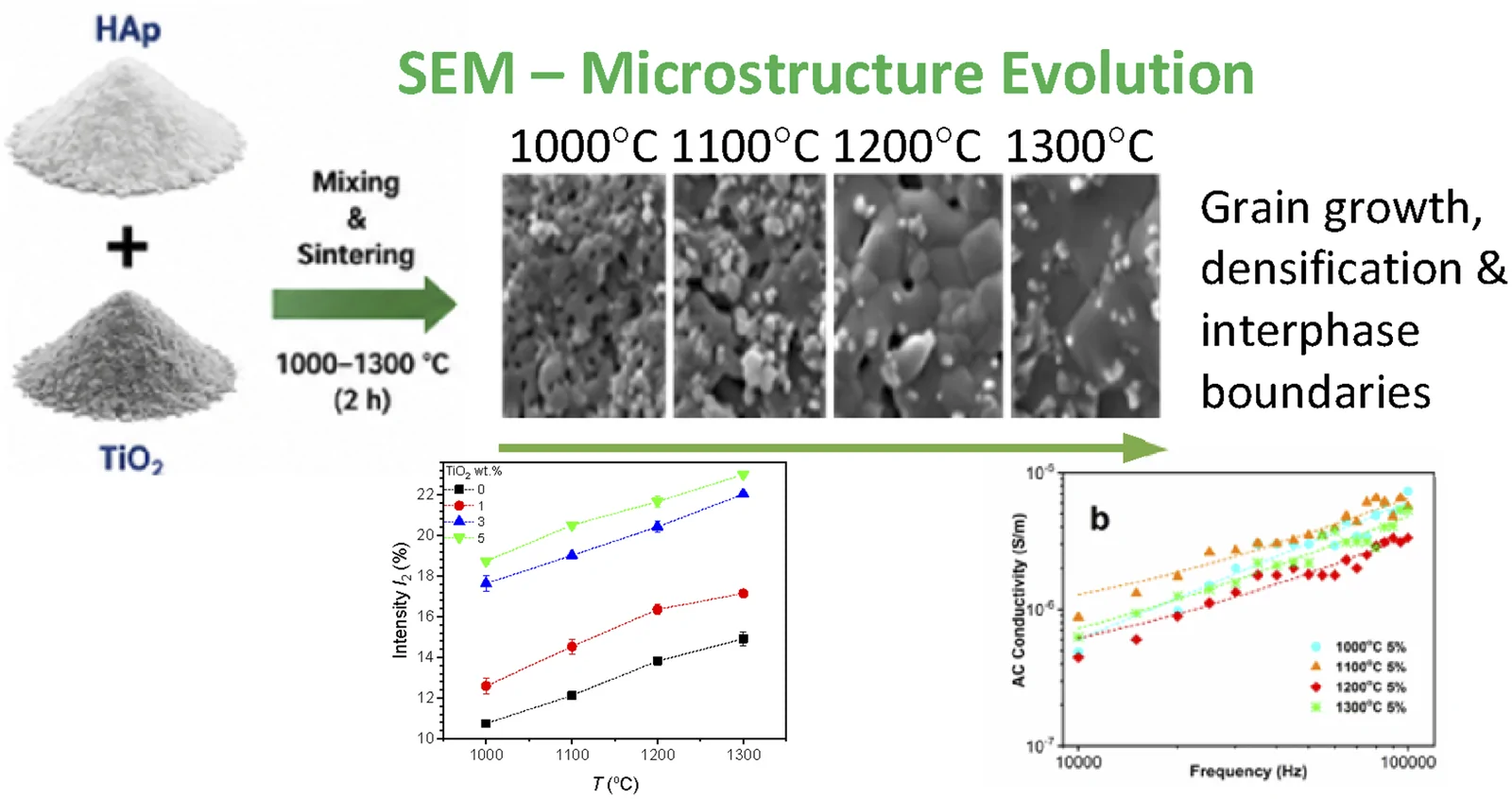

## 1 Introduction

Hydroxyapatite (HAp) is one of the most extensively studied calcium phosphate ceramics because of its close chemical similarity to the mineral component of bone and its excellent biocompatibility.^1^ Beyond biomedical applications, HAp has attracted considerable interest as a functional ceramic whose structural, mechanical, and electrical properties can be tailored through compositional modification and thermal processing. Various oxide additives, including alumina, zirconia, and titania (TiO_2_), have therefore been incorporated into HAp to improve its densification behavior, mechanical performance, and functional properties.^2–7^ Among these additives, TiO_2_ has received particular attention owing to its chemical stability and strong interaction with calcium phosphate phases during sintering.

When HAp is combined with TiO_2_, the resulting system undergoes complex structural and chemical evolution during sintering. Previous studies have shown that HAp–TiO_2_ mixtures can undergo partial decomposition of HAp into β-tricalcium phosphate (β-TCP), accompanied by solid-state reactions between the calcium phosphate and TiO_2_ phases that lead to the formation of calcium titanates such as CaTiO_3_ at elevated temperatures.^8–10^ These transformations depend strongly on sintering temperature, TiO_2_ content, Ca/P ratio, and solid-state diffusion processes, and can significantly influence densification, microstructure, and electrical properties. Despite considerable progress in understanding vacancy-type defects in HAp-based materials,^11–15^ the evolution of nanoscale defect structures during the transformation of HAp–TiO_2_ mixtures into multiphase calcium phosphate–titanate ceramics remains insufficiently understood, particularly regarding the relationships among phase evolution, interfacial defect formation, and electrical behavior.

**Fig. 1:**
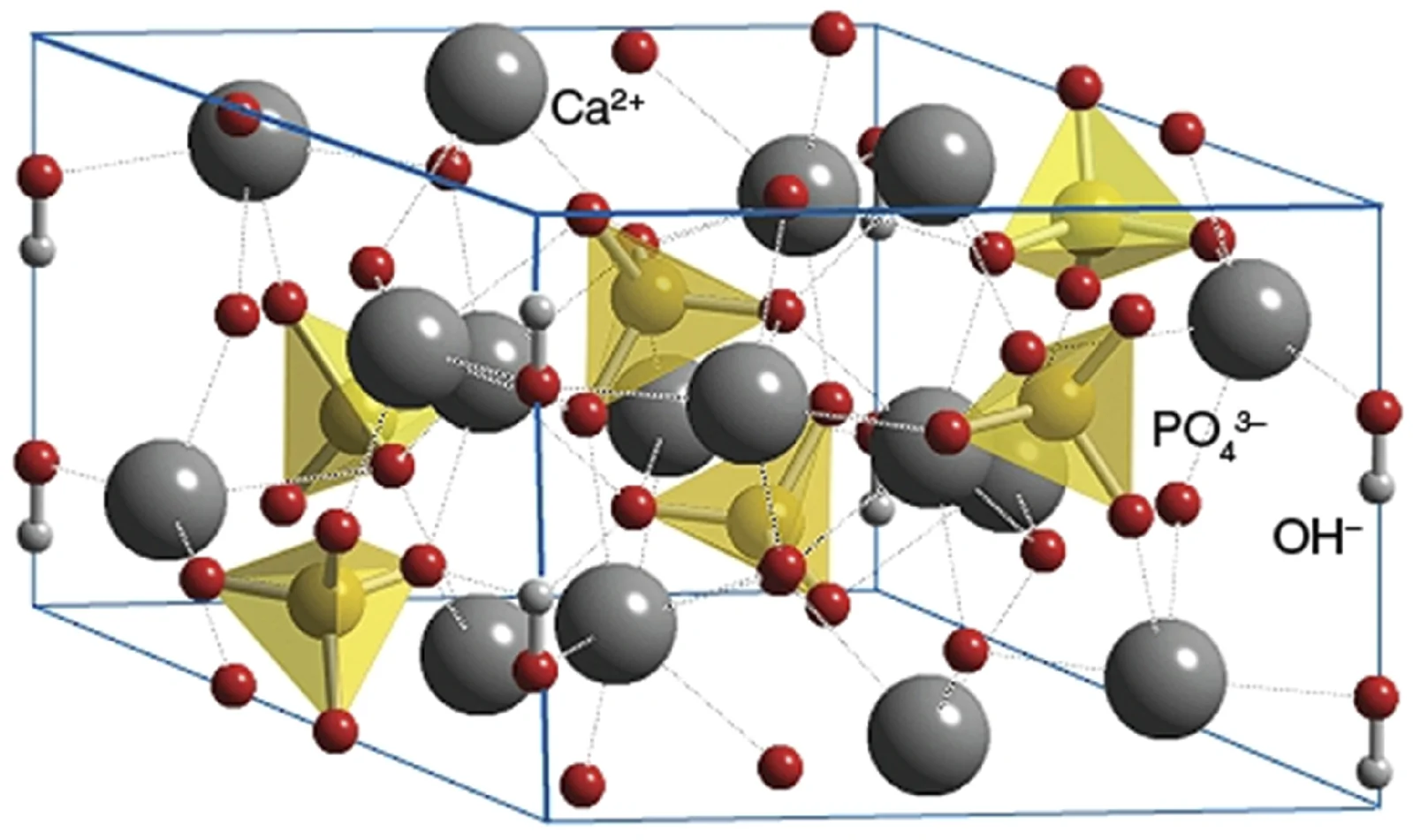
Molecular configuration of a hexagonal HAp cell taken from ref. 29 (spheres in red: O, small gray: H, large gray: Ca, and yellow: P).

Defect engineering plays a crucial role in controlling the structural, mechanical, and electrical properties of HAp-based ceramics.^16^ Vacancy-type defects influence densification, lattice stability, grain-boundary structure, and charge transport. In HAp, energetically favorable defects include calcium vacancies 
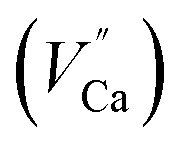
, hydroxyl vacancies 
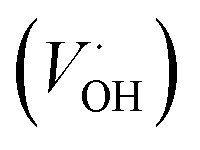
, and oxygen-related defects, which can form coupled defect complexes and vacancy chains along the crystallographic *c*-axis (Fig. 1).^12,13,17–20^ While conventional characterization techniques such as XRD and SEM provide indirect information on structural changes, they are limited in resolving vacancy-type defects at the atomic scale. In contrast, positron annihilation lifetime spectroscopy (PALS) offers a sensitive, non-destructive probe for detecting open-volume defects and their evolution during processing.^14,21,22^

The incorporation of TiO_2_ modifies the defect landscape through phase interactions, interfacial formation, lattice distortion, and chemical heterogeneity, leading to complex defect environments that cannot be fully resolved by conventional characterization techniques alone. In particular, the formation of Ti-containing secondary phases and associated interphase boundaries may introduce additional structural heterogeneity and localized defect environments that can influence charge transport in multiphase calcium phosphate ceramics.^23^ More generally, the electrical response of calcium phosphate ceramics is sensitive to phase composition, grain boundaries, defect structure, porosity, and thermal processing, all of which may affect charge-transport and polarization behavior.^24–27^

In this study, the structural, microstructural, and defect evolution of HAp–TiO_2_-derived calcium phosphate–titanate ceramics (THAp) is systematically investigated using X-ray diffraction (XRD), scanning electron microscopy (SEM), positron annihilation lifetime spectroscopy (PALS), and electrical conductivity measurements. Particular emphasis is placed on correlating phase evolution, interfacial defect formation, and electrical transport through a combined structural and positron annihilation analysis. This integrated approach provides new insight into the relationships among processing conditions, phase evolution, defect chemistry, and electrical behavior in HAp–TiO_2_-derived calcium phosphate–titanate ceramics.

## 2 Materials and methods

### 2.1 HAp and TiO_2_ materials and their composites

HAp with less than 0.7% impurity (99.3% pure) was purchased from Acros Organics, and titanium(iv) oxide was purchased from Rediel-de Haë. HAp powder was first diluted for nearly 1 hour *via* sonic vibration to eliminate agglomerations due to humidity. The particle size analysis was performed with a particle/zeta sizer (Malvern Panalytical, Zetasizer ver. 8.02), and the corresponding results are provided in Fig. S1(a) and (b) in the SI. The average particle sizes of both powders are close to each other (697.8 nm and 634.2 nm) at the nanoscale. Grinding and mixing were performed using glass and plastic bottles containing HAp, TiO_2_, alumina balls (99.5% pure alumina spheres, 5 mm diameter), and ethyl alcohol for approximately 1.3 hours (at a rotational speed of 19.93 cm min^−1^).

HAp has a hexagonal structure with lattice parameters of *a* = *b* = 0.9432 nm and *c* = 0.6101 nm, as shown in Fig. 1. The Ca/P ratio is 1.67. The nonporous density of hydroxyapatite is approximately 3.16 g cm^−3^, and the approximate microhardness is approximately 500–600 HV. The apatite structure is open to substitution by other ions. Ions replacing the Ca^+2^, PO_4_^−3^ and OH^−^ groups in the apatite structure cause changes in the lattice parameters, morphology, and solubility properties of the structure without substantially changing the hexagonal symmetry.^28^

HAp powder was mixed with titania powder at weight percentages of 1, 3, and 5%. The mixture was processed through ball milling in a polyethylene jar that contained 5 mm diameter alumina media and alcohol. The resulting slurry, which included alumina media, occupied nearly two-thirds of the jar's volume. Milling was performed for 1.3 hours at a rotational speed of 20 cm min^−1^. Following the milling process, the slurry was separated from the media and dried for 1 hour at 150 °C in a drying furnace (Binder) under ambient conditions. The powder mixtures were then compressed *via* hardened steel molds in accordance with British Standard 7253. A unidirectional press applied a pressure of 10 MPa for approximately 3 minutes. The resulting pellets had an average diameter of 7 mm and a height of 10 mm. The composite pellets subsequently underwent a sintering process at temperatures of 1000, 1100, 1200, and 1300 °C for 3 hours under normal atmospheric conditions in a box furnace (Nabertherm HT16-18). The hardness of the pellets was evaluated *via* a microhardness testing apparatus.

### 2.2 Instruments

XRD analysis was conducted *via* a Bruker D2 Phaser instrument, with parameters set to Cu Kα = 1.542 Å, 30 kV, 10 mA, a step scan of 0.01°, and a 2*θ* range of 10° to 80°. SEM analysis was performed *via* a Zeiss EVO MA10 SEM, and the samples were coated with gold *via* a Quorum SC7620 PVD apparatus. Microhardness measurements of the sintered samples were conducted *via* a Vickers microhardness tester, the FutureTech FM-ARS 7000, with a force of 0.5 N applied to the diamond indenter.

### 2.3 Positron annihilation lifetime spectroscopy (PALS)

PALS uses a fast–fast coincidence setup system to measure the time interval between the birth signal, a 1274 keV prompt gamma, and the stop signal, a 511 keV positron annihilation gamma.^30,31^ For the detection of both gamma rays, the setup included two plastic scintillators (BC422) paired with photomultiplier tubes (Hamamatsu R2059) and an ORTEC base. Furthermore, two constant fractional differential discriminators (ORTEC 583B) and a time-to-amplitude converter (ORTEC 266) were connected to the MCA time spectrum (ORTEC ASPEC 927) to provide timing signals and information. The LT9.2 code program^32^ was utilized to extract the lifetimes and their intensities from the spectrum. The LT code is a well-regarded analytic tool used to accurately model PALS data deconvoluted from the lifetime spectrum into several exponential components.^32^

PALS experiments were conducted using two samples, each with a thickness greater than 2 mm, which encased a positron source containing approximately 30 µCi of ^22^NaCl on a thin aluminum foil (6 µm thick).^33,34^ Source correction was applied with a resolution of approximately 350 ps. A total of one million counts were recorded for each sample measurement.

The analyzed spectra yield two distinct lifetime components corresponding to different annihilation environments. The shorter lifetime component (*τ*_1_, with intensity *I*_1_) is attributed to positron annihilation in bulk-like regions of the HAp matrix, slightly influenced by shallow defects. The longer lifetime component (*τ*_2_ with intensity *I*_2_) is associated with positron trapping at open-volume defects, including vacancy-type defects, grain boundaries, and interfacial regions formed between HAp and TiO_2_ phases. It should be noted that the overlap of lifetime contributions from different defect types and microstructural features makes the unambiguous separation of individual annihilation sites challenging.^35^

### 2.4 Electrical measurements

To perform electrical measurements, the HAp and THAp pellets were positioned between two identical metal masks featuring 3 mm diameter holes. Circular silver electrodes were deposited on each side of the pellets in a high vacuum environment (less than 10^−6^ mbar) *via* the Edwards Auto 500 thermal evaporation system. The silver used had a purity of 99.99%. Silver paste was used to make electrical contacts between the conducting wire and the silver electrodes.

All electrical measurements of the pellets were conducted in a vacuum environment (10^−2^ mbar) and in the dark. The DC conductivity values were derived from the *I*–*V* curves of both the HAP and THAp pellets. The voltage across each side of the pellets, along with the corresponding current values, was recorded by applying sweep DC voltages ranging from −1.0 to 1.0 V at 25 °C *via* a Keithley model 6517B electrometer. Additionally, the electrical response of the pellets to the AC signal was examined as a function of frequency. AC conductivity measurements were performed in a vacuum environment across frequencies from 40 to 10^4^ Hz at 25 °C using a Keithley model 3330 LCZ meter.

## 3 Results and discussion

### 3.1 XRD results

**Fig. 2:**
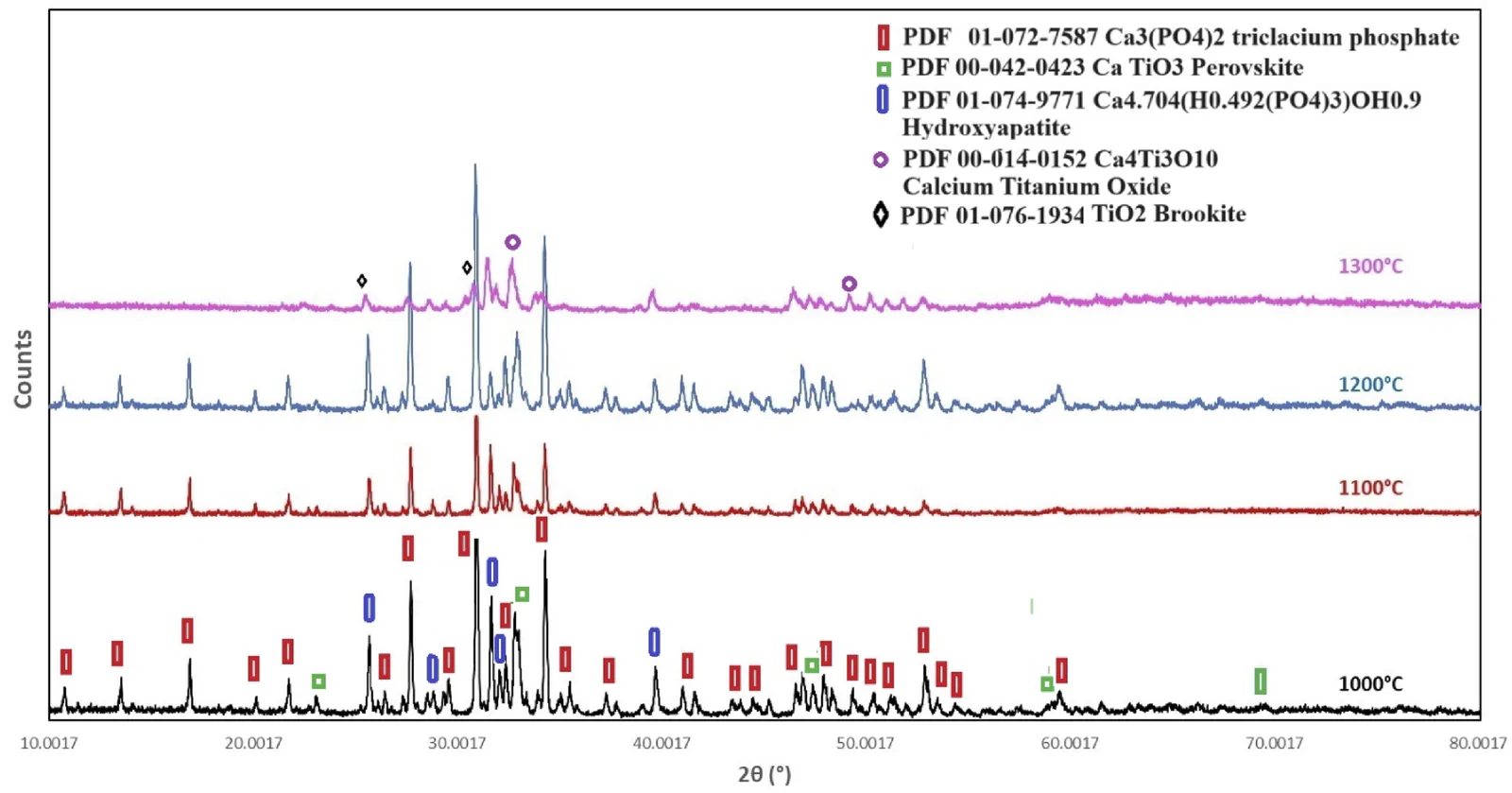
XRD analysis of the HAp composite samples with 5 wt% TiO_2_ added was performed at sintering temperatures of 1000, 1100, 1200, and 1300 °C.

Fig. 2 presents the XRD patterns of HAp composites containing 5 wt% TiO_2_ sintered at 1000, 1100, 1200, and 1300 °C. The diffraction profiles reveal a progressive transformation of the initial HAp phase with increasing temperature. The most prominent reflections at 2*θ* ≈ 27.72°, 31.01°, and 34.30° are attributed to whitlockite-type calcium phosphate (Ca_3_(PO_4_)_2_, PDF: 01-072-7587), indicating the formation of β-TCP as the dominant phase at elevated temperatures.

At 1000 °C, weak reflections near 2*θ* ≈ 31.6° suggest the presence of a minor residual HAp-related phase. However, considering the known thermal instability of nonstoichiometric HAp phases containing HPO_4_ groups above about 700 °C, these reflections are more appropriately assigned to residual or partially decomposed HAp rather than a stable HPO_4_-containing phase. The contribution of this phase decreases progressively with increasing sintering temperature.

With further temperature increase, additional reflections corresponding to calcium titanate (CaTiO_3_, PDF: 00-042-0423) emerge, particularly above 1100 °C, with characteristic peaks near 2*θ* ≈ 32.99° and 47.44°. This indicates that TiO_2_ participates in solid-state reactions with calcium phosphate phases, leading to the formation of titanate compounds. The reaction can be qualitatively described by:^10,29^Ca_10_(PO_4_)_6_(OH)_2_ + TiO_2_ → 3Ca_3_(PO_4_)_2_ + CaTiO_3_ + H_2_O↑although the exact reaction pathway is expected to proceed through intermediate phases and diffusion-controlled processes.

The calcium phosphate system exhibits polymorphic transformations, where β-TCP is stable at room temperature, while α-TCP begins to form above about 1100 °C. During cooling, α-TCP reverts to β-TCP, resulting in the observed dominance of β-TCP in the final microstructure, which is characterized by a tetragonal crystal structure with lattice parameters of *a* = 10.40 Å and *c* = 37.38 Å.

**Fig. 3:**
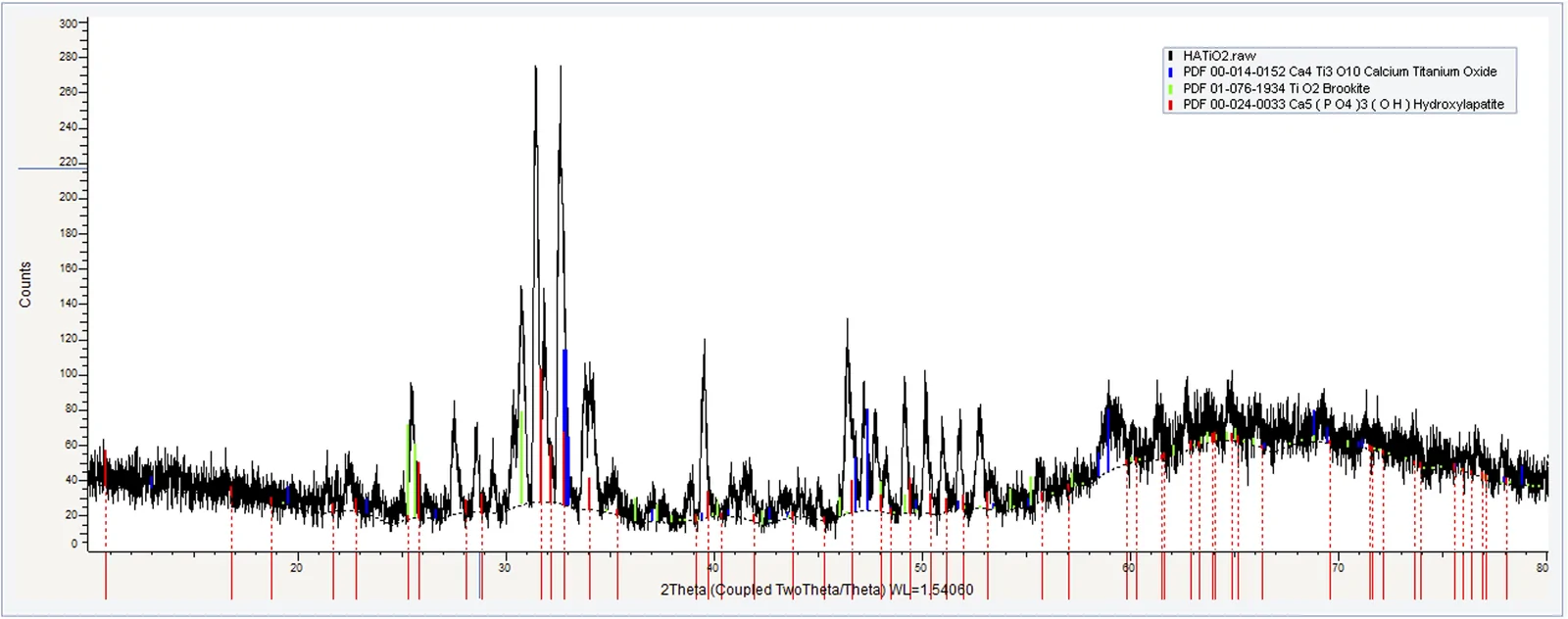
XRD results for the 5 wt% TiO_2_ composite HAp sintered at 1300 °C for 3 hours.

Fig. 3 shows a detailed XRD pattern of the sample sintered at 1300 °C for 3 hours. In addition to the dominant β-TCP and CaTiO_3_ phases, additional reflections near 2*θ* ≈ 33° are consistent with the presence of a layered calcium titanate phase such as Ca_4_Ti_3_O_10_ (PDF: 00-014-0152), with lattice parameters of *a* = 5.44 Å, *b* = 5.53 Å, and *c* = 27.18 Å.^8^ However, owing to the partial overlap of diffraction peaks with those of calcium phosphate and other titanate phases, this phase assignment should be regarded as tentative and is based primarily on PDF matching. Unambiguous identification would require complementary analyses, such as quantitative Rietveld refinement or high-resolution transmission electron microscopy with selected-area electron diffraction (SAED), which are beyond the scope of the present study.

Weak residual TiO_2_ reflections (brookite, PDF: 01-076-1934) may still be detected, suggesting that TiO_2_ is largely consumed but not necessarily completely eliminated in all regions of the sample.

The progressive increase in phase complexity observed in the XRD patterns reflects the coexistence of β-TCP and Ti-containing phases, indicating extensive solid-state reactions between HAp and TiO_2_ during sintering. Similar behavior has been widely reported in calcium phosphate-based ceramic systems, where the incorporation of secondary oxide phases promotes phase decomposition, multiphase formation, and structural heterogeneity.^36–38^

In particular, studies on THAp composites have demonstrated that TiO_2_ exhibits a strong thermodynamic tendency to diffuse into the HAp lattice and react to form calcium titanate (CaTiO_3_), accompanied by a reduction or disappearance of the initial TiO_2_ phase at elevated temperatures. This process is indicative of significant chemical interaction between the constituent phases and leads to a redistribution of calcium and titanium within the microstructure.^8^

Furthermore, the decomposition of hydroxyapatite into β-tricalcium phosphate (β-TCP) and related phases is commonly observed in composite systems containing secondary ceramic additives, particularly under high-temperature sintering conditions. These transformations are driven by interfacial reactions and compositional instability and are often accompanied by the formation of multiphase microstructures.^39^ In addition, the formation of CaTiO_3_ has been reported to influence the phase stability and structural evolution of calcium phosphate systems, contributing to enhanced phase interaction and chemical heterogeneity.^39^

As a consequence, such multiphase systems inherently introduce lattice distortions, residual stresses, and defect-rich regions, particularly at phase boundaries and interfacial zones. Moreover, previous studies have shown that incomplete reactions and phase transformations in ceramic composites may also lead to the formation of non-crystalline or poorly ordered regions, as well as microstructural defects such as pores and microcracks.^36–38^

Therefore, the present XRD results, revealing the coexistence of TCP, CaTiO_3_, and residual TiO_2_ phases, strongly indicate the development of a chemically heterogeneous and structurally complex microstructure. Although XRD does not directly resolve vacancy-type defects or open-volume porosity, the formation of such multiphase assemblages is widely associated with increased defect density and heterogeneous defect distribution, which are further elucidated by PALS analysis.

Although the present XRD analysis clearly establishes the phase evolution occurring during sintering, including the transformation of HAp into β-TCP and the formation of calcium titanate phases, quantitative phase fractions and refined lattice parameters were not determined by Rietveld refinement. Such crystallographic analysis would provide valuable additional information on the relative phase contents, structural distortion, and phase evolution, and will therefore be considered in future work.

### 3.2 SEM/EDS analysis results

**Fig. 4:**
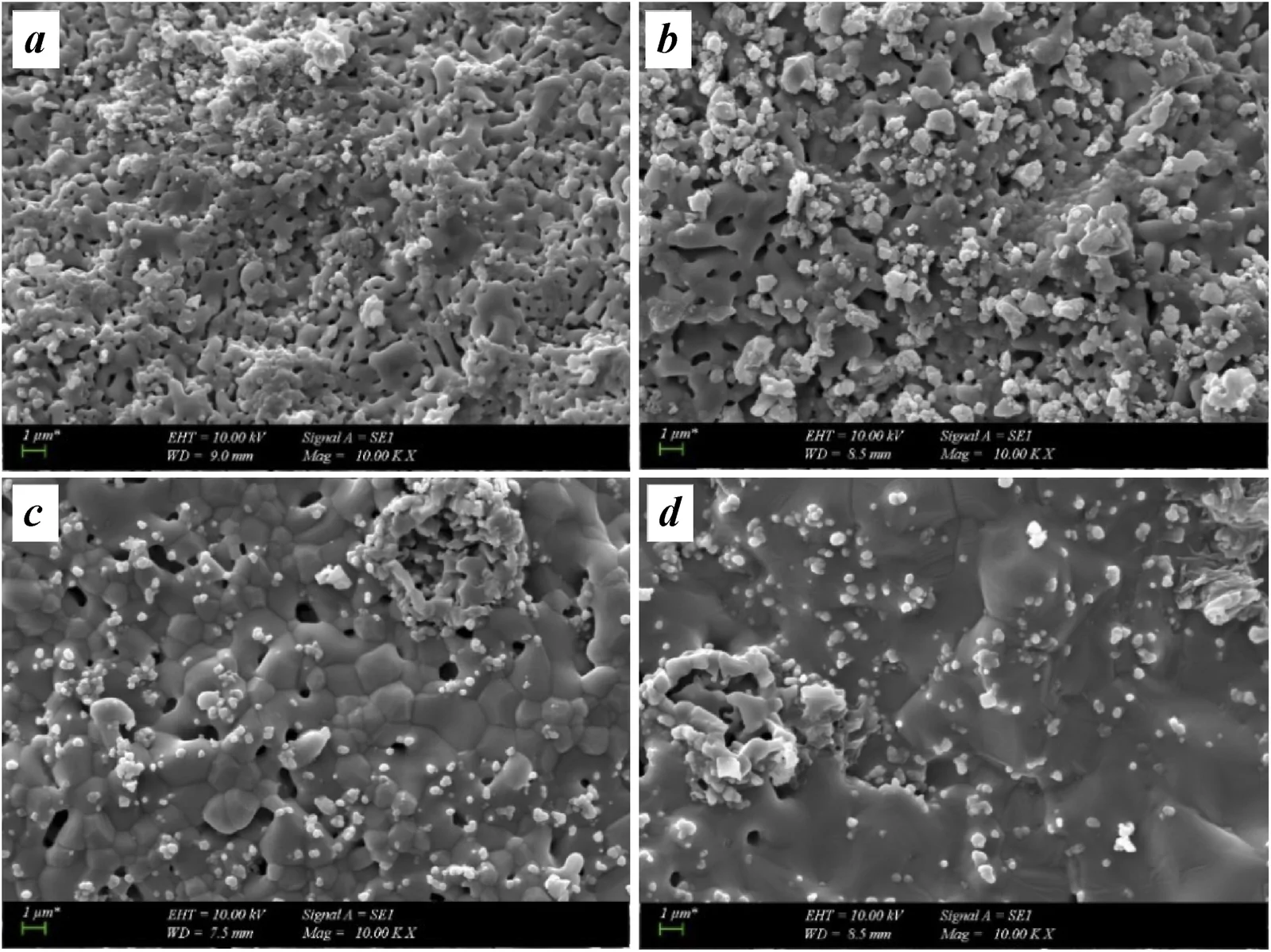
SEM micrographs of the HAp samples in the SEI with 5% titania sintered at (a) 1000 °C, (b) 1100 °C, (c) 1200 °C, and (d) 1300 °C for 1 hour at 10 Kx.

Fig. 4 shows SEM micrographs of HAp samples containing 5 wt% TiO_2_ sintered at 1000, 1100, 1200, and 1300 °C for 1 h. The corresponding BEI and lower-magnification images are provided in the SI. A highly porous and weakly consolidated microstructure is observed at 1000 °C (Fig. 4a), indicating incomplete sintering and limited particle coalescence. The grains remain relatively fine and the interparticle contacts are poorly developed, consistent with an early stage of solid-state sintering. At 1100 °C (Fig. 4b), neck formation between adjacent particles becomes more evident, indicating enhanced mass transport and progressive densification. However, isolated irregular pores are still present, suggesting that sintering remains incomplete.

At 1200 °C (Fig. 4c), the microstructure becomes more compact, with a marked reduction in open porosity and improved packing of the grains. The grains appear more uniform and better bonded, indicating a more advanced sintering stage. From a microstructural point of view, this temperature provides the best balance between densification and grain coarsening among the samples examined. At 1300 °C (Fig. 4d), the surface becomes smoother and grain growth is more pronounced. Localized dense regions and morphological changes suggest enhanced grain-boundary mobility and possible liquid-assisted mass transport. However, this interpretation remains tentative on the basis of SEM alone. In combination with the XRD results, the microstructural evolution at this temperature is consistent with extensive phase interaction and the formation of a multiphase calcium phosphate–titanate system rather than a single-phase HAp ceramic.

Overall, the SEM observations confirm a clear temperature-dependent evolution from a porous and weakly sintered structure at 1000 °C to a denser microstructure at 1200 °C, followed by pronounced coarsening at 1300 °C. Thus, among the investigated sintering temperatures, 1200 °C produced the most balanced microstructure, characterized by high densification together with a relatively uniform grain morphology and limited grain coarsening. This interpretation is supported by the combined density measurements (Fig. 6b) and SEM observations (Fig. 4).

EDS analysis of the sample sintered at 1300 °C, presented in the SI, confirms the presence of Ca, P, O, and Ti within the analyzed region, demonstrating the coexistence of Ti-containing phases and calcium phosphate phases in the sintered composite. However, EDS alone cannot determine whether Ti is present as residual TiO_2_, calcium titanate, or as a species incorporated into the calcium phosphate lattice. Likewise, local Ca/P ratios obtained from EDS should be interpreted with caution, as they do not, by themselves, establish the phase stability of HAp after high-temperature sintering. Therefore, in the present study, EDS is used solely to confirm the elemental composition, whereas phase identification is based primarily on XRD.

It should also be noted that EDS provides only semi-quantitative elemental information and cannot reliably distinguish between Ti-containing crystalline phases with similar elemental compositions, such as TiO_2_ and calcium titanate phases. Furthermore, owing to the interaction volume of the electron beam and the overlap of characteristic X-ray signals, EDS is not suitable for accurate phase quantification or determination of local stoichiometry in multiphase ceramics. Nor can it provide information on vacancy concentrations or defect populations. Consequently, phase identification in the present work relies primarily on XRD analysis, whereas vacancy-type defects are characterized by PALS.

### 3.3 PALS results and evaluation

**Fig. 5:**
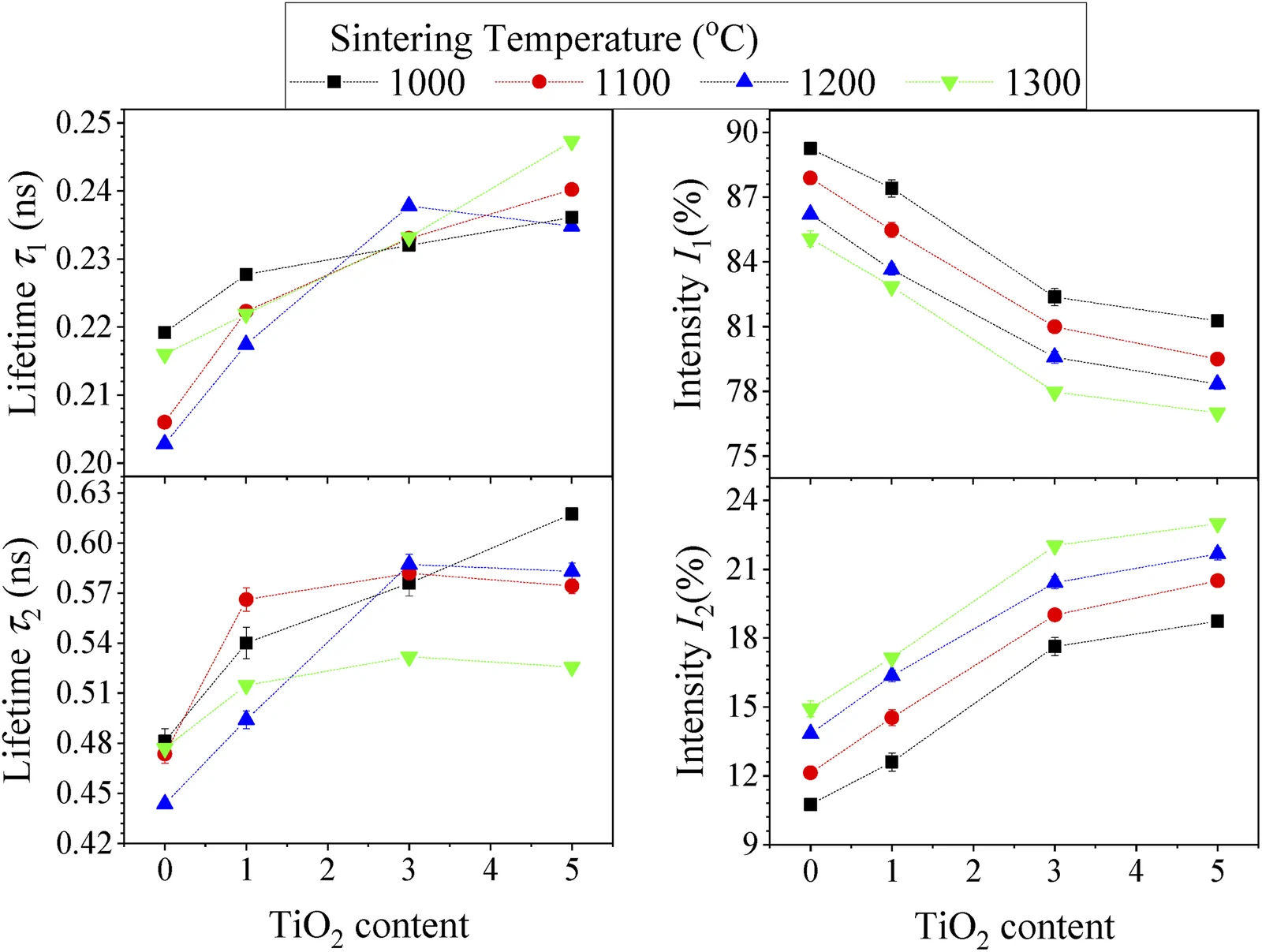
Positron annihilation lifetime parameters: lifetimes *τ*_1_ and *τ*_2_, and their intensities *I*_1_, and *I*_2_*versus* TiO_2_ contents.

The evolution of defect structure in THAp composites as a function of TiO_2_ content and sintering temperature was investigated using PALS. The extracted lifetimes (*τ*_1_, *τ*_2_) and their corresponding intensities (*I*_1_, *I*_2_) are summarized in Table S2 and presented in Fig. 5a–d as a function of TiO_2_ content for the different sintering temperatures and in Fig. S9(a–d) as a function of the sintering temperatures.

The PALS spectra were satisfactorily resolved into two lifetime components. Attempts to introduce an additional lifetime component did not produce statistically stable fitting parameters because of the limited instrumental time resolution (∼350 ps) and the substantial overlap between annihilation components. Consequently, the two-component model provided the most reliable and physically meaningful description of the spectra. The short lifetime component, *τ*_1_ (0.20–0.25 ns), is attributed to positron annihilation in bulk-like regions of HAp, slightly perturbed by shallow defects and lattice distortions. The second lifetime component, *τ*_2_ (0.44–0.62 ns), is attributed to positron trapping at a combination of open-volume defects, including vacancy clusters, grain boundaries, interphase boundaries, and interfaces between the calcium phosphate and calcium titanate phases. This interpretation follows the diffusion-reaction model proposed by Brunner and Würschum^35^ and is supported by the concurrent XRD and SEM observations. The absence of a distinct long-lived positronium component indicates that large mesoscopic pores are limited, particularly at elevated sintering temperatures, consistent with the dense microstructures observed by SEM. In the present ceramic system, positronium formation is expected to be negligible, and the observed *τ*_2_ therefore originates predominantly from trapped positrons rather than positronium annihilation. Consequently, direct conversion of *τ*_2_ into an equivalent free-volume radius using the Tao–Eldrup model is not considered appropriate for the present dense ceramic system. Instead, *τ*_2_ is interpreted qualitatively as reflecting changes in the characteristic size and local environment of open-volume defects.

The variation of PALS parameters with sintering temperature reveals a clear densification-driven evolution of the defect structure. As shown in Fig. S9, *τ*_2_ generally decreases with increasing temperature, especially above 1100–1200 °C, indicating the progressive elimination or shrinkage of large open-volume defects. This behavior is in excellent agreement with SEM observations, where the microstructure evolves from loosely packed grains with significant porosity at 1000 °C to a dense and well-sintered structure at 1300 °C characterized by grain growth, neck formation, and reduced porosity. Despite the reduction in *τ*_2_, the intensity *I*_2_ increases systematically with temperature, accompanied by a decrease in *I*_1_. This indicates that although large defects diminish in size, positron trapping becomes more efficient due to defect redistribution. Grain growth increases the relative contribution of grain boundary regions, while defects migrate toward energetically favorable interfacial configurations, leading to a higher probability of positron trapping. Consequently, the defect structure evolves from a pore-dominated regime at low temperatures to an interface-controlled regime at high temperatures.

A similarly systematic trend is observed with increasing TiO_2_ content, as shown in Fig. 5. The gradual increase in *τ*_1_ suggests that TiO_2_ incorporation induces lattice distortion and modifies the local electronic environment in the HAp matrix. This effect can be attributed to structural mismatch and defect formation associated with Ti-containing phases and interfacial strain. More significantly, *τ*_2_ and *I*_2_ increase markedly with TiO_2_ content, indicating enhanced positron trapping at open-volume defects. This behavior reflects the formation of additional defect sites associated with THAp interfaces, grain boundaries, and vacancy-type defects generated during composite processing. The concurrent decrease in *I*_1_ further confirms the transition from bulk-like annihilation to defect-dominated annihilation processes.

The defect formation mechanisms can be rationalized using Kröger–Vink notation. During high-temperature sintering, hydroxyapatite undergoes dehydroxylation, which can be expressed as 
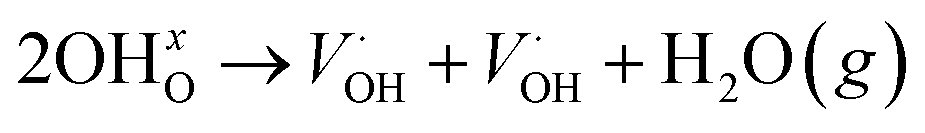
. The resulting positively charged hydroxyl vacancies are compensated by the formation of doubly negatively charged calcium vacancies according to 
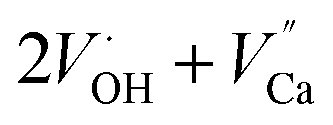
 defect cluster, leading to a non-stoichiometric composition of the form Ca_10−*x*_(PO_4_)_6_(OH)_2−*x*_. The introduction of TiO_2_ further modifies the defect chemistry through phase interaction and cation redistribution. At elevated temperatures, TiO_2_ reacts with calcium-containing species to form CaTiO_3_, as confirmed by XRD and EDS analyses. This process can be described by reactions such as 
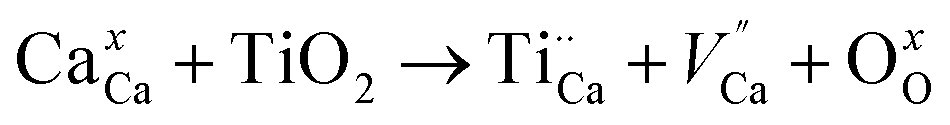
, which introduce calcium vacancies and alter the local charge balance. The formation of CaTiO_3_ and related phases leads to the development of heterogeneous interfaces and defect-rich regions, which act as efficient positron trapping centers. These observations are consistent with increased positron trapping at vacancy-type defects, grain boundaries, and interfacial regions generated during phase evolution. Although PALS is highly sensitive to open-volume defects, complementary techniques such as XPS, Raman spectroscopy, and TEM would provide additional confirmation of the chemical nature and spatial distribution of these defect structures.

The microstructural observations further support this interpretation. SEM images show that TiO_2_ particles, initially dispersed at lower temperatures, become increasingly embedded within the HAp matrix at higher temperatures, resulting in stronger interfacial contact and reduced porosity. Simultaneously, XRD and SEM results confirm the formation of secondary phases, indicating significant chemical interaction between HAp and TiO_2_. These structural and chemical changes contribute to the redistribution of defects from large pores toward smaller, more uniformly distributed interfacial trapping sites. As a result, the defect landscape evolves from large open-volume defects at low temperatures to smaller but more numerous defects localized at grain boundaries and interfaces at higher temperatures.

Overall, the combined PALS, SEM, and XRD analyses demonstrate that the defect structure of THAp composites is governed by a complex interplay between densification, phase evolution, and interfacial interactions. While increasing temperature reduces the size of open-volume defects through densification, TiO_2_ addition enhances defect formation and interfacial trapping. This leads to a transition from pore-controlled to interface-controlled defect structures, which is sensitively captured by the evolution of positron lifetime parameters. These findings highlight the critical role of interfacial defect chemistry in determining the microstructural and functional properties of HAp-based composites.

### 3.4 Hardness and density measurements

**Fig. 6:**
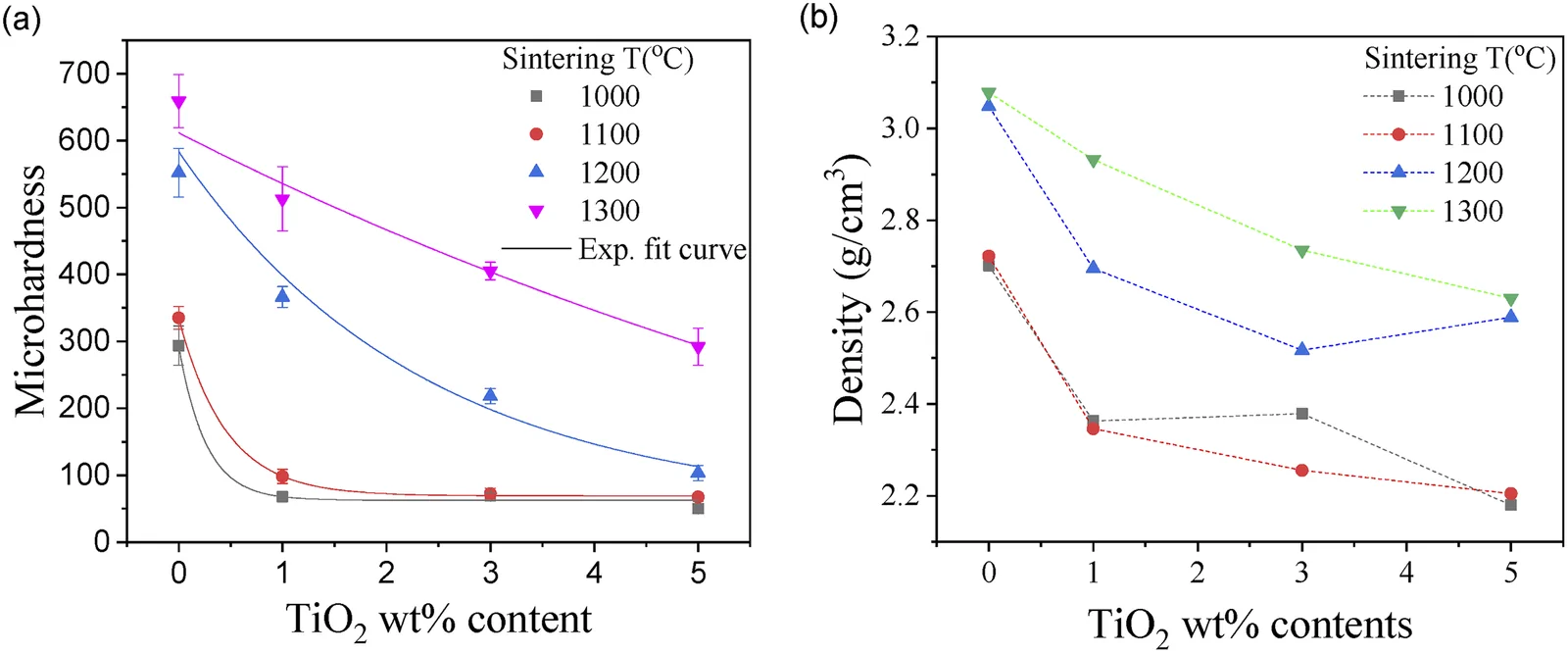
(a) The microhardness and (b) the density of THAp as a function of the weight percentage (wt%) of TiO_2_ at the indicated sintering temperatures.

Microhardness measurements were performed using a load of 0.5 N applied through a diamond indenter. At least three indentations were taken for each sample, and the average values with corresponding standard deviations are summarized in Table S1. The variation of hardness as a function of TiO_2_ content at different sintering temperatures is presented in Fig. 6(a).

In general, the hardness increases with increasing sintering temperature for all compositions, which can be attributed to enhanced densification and improved interparticle bonding. This trend is particularly evident in pure HAp samples, where the progressive elimination of porosity and grain boundary consolidation leads to a continuous increase in hardness.

In contrast, the effect of TiO_2_ addition on hardness depends strongly on both composition and sintering temperature. At lower temperatures (1000 and 1100 °C), the incorporation of TiO_2_ results in only minor changes in hardness, suggesting that the microstructure remains dominated by incomplete sintering and limited phase interaction. However, at higher temperatures (1200 and 1300 °C), a pronounced decrease in hardness is observed with increasing TiO_2_ content. This reduction in hardness can be correlated with the phase evolution and microstructural changes identified by XRD and SEM analyses. Above ∼1100 °C, hydroxyapatite undergoes partial decomposition into β-tricalcium phosphate (β-TCP), while TiO_2_ reacts with calcium-containing species to form calcium titanate (CaTiO_3_). The formation of these secondary phases results in a multiphase microstructure characterized by increased interfacial area and chemical heterogeneity. Such heterogeneity can introduce residual stresses and defect-rich regions, particularly at phase boundaries, which act as mechanically weaker zones under indentation.

Furthermore, PALS results indicate an increase in defect-related positron trapping with TiO_2_ addition, suggesting a higher density of vacancy-type defects and interfacial trapping sites. These defects can reduce the effective load-bearing capacity of the material, contributing to the observed decrease in hardness. Therefore, the reduction in hardness at higher TiO_2_ contents is not solely due to phase composition, but rather to the combined effects of defect generation, interfacial structure, and microstructural heterogeneity.

Overall, while increasing sintering temperature enhances densification and hardness in pure HAp, the introduction of TiO_2_ at elevated temperatures leads to a more complex microstructure in which defect accumulation and phase interactions outweigh the benefits of densification, resulting in a reduction in hardness.

The variation of density as a function of TiO_2_ content and sintering temperature is shown in Fig. 6(b). For all sintering temperatures, the density decreases systematically with increasing TiO_2_ content, indicating that TiO_2_ addition hinders densification of the HAp matrix. This behavior can be attributed to the formation of a multiphase microstructure, as confirmed by XRD, where HAp partially decomposes into β-TCP and reacts with TiO_2_ to form CaTiO_3_ and related phases. The presence of these phases introduces chemical heterogeneity and increases the volume fraction of interfacial regions, which act as barriers to mass transport during sintering and limit densification. SEM observations further support this interpretation, showing that TiO_2_ addition leads to a less uniform microstructure and the persistence of residual porosity. As expected, density increases with sintering temperature due to enhanced diffusion, grain growth, and pore elimination. However, even at higher temperatures, the negative effect of TiO_2_ on densification persists, indicating that phase interaction and interfacial constraints dominate over thermal densification. These results are consistent with PALS measurements, where increased TiO_2_ content leads to enhanced positron trapping, reflecting a higher density of open-volume defects and interfacial trapping sites. The combined results demonstrate that TiO_2_ addition promotes structural heterogeneity and defect formation, which adversely affect densification behavior.

### 3.5 DC conductivity studies

All electrical measurements were performed at room temperature in order to isolate the influence of phase evolution, microstructure, and defect structure on the electrical properties while avoiding additional contributions arising from thermally activated charge transport. This approach enables a direct comparison of the effects of TiO_2_ content and sintering temperature under identical measurement conditions.

**Fig. 7:**
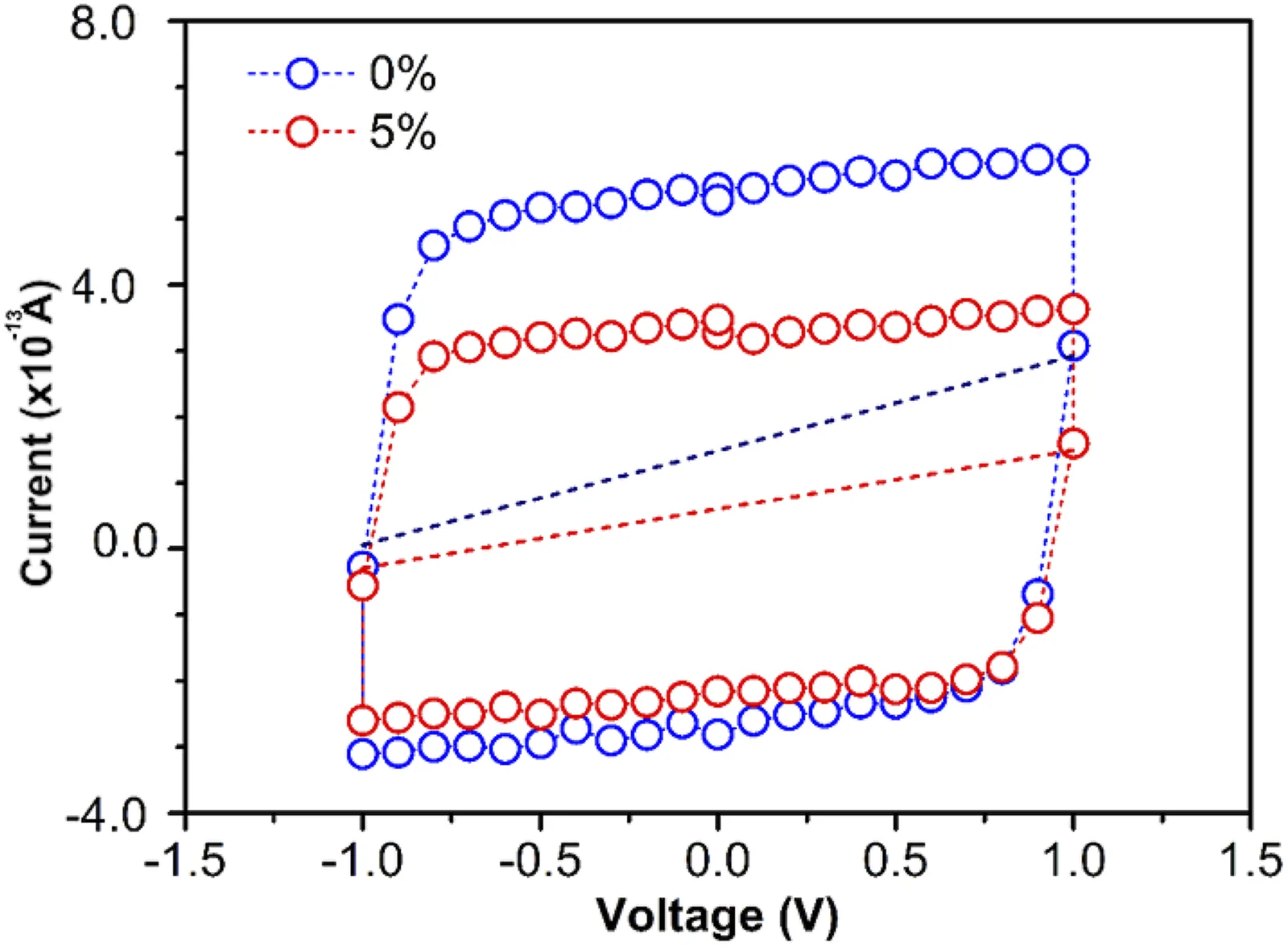
*I*–*V* curves of the undoped and 5 wt% TiO_2_-doped pellets sintered at 1200 °C.

Fig. 7 shows representative *I*–*V* characteristics of the pellets sintered at 1200 °C for different TiO_2_ contents with the trend lines of the curves. Similar behavior was observed for samples sintered at other temperatures. The *I*–*V* curves are predominantly linear, indicating ohmic conduction; however, a clear hysteresis between forward and reverse sweeps is present. The observed hysteresis is attributed to charge trapping and detrapping processes associated with defect states and interfacial regions within the composites. In THAp systems, these trapping centers arise from vacancy-type defects, grain boundaries, and phase interfaces, as identified by PALS measurements. During voltage cycling, charge carriers are temporarily captured by these defect sites and subsequently released, leading to delayed current response and the formation of hysteresis loops. The magnitude of hysteresis therefore reflects the density and distribution of defect-related trapping sites within the material.

**Fig. 8:**
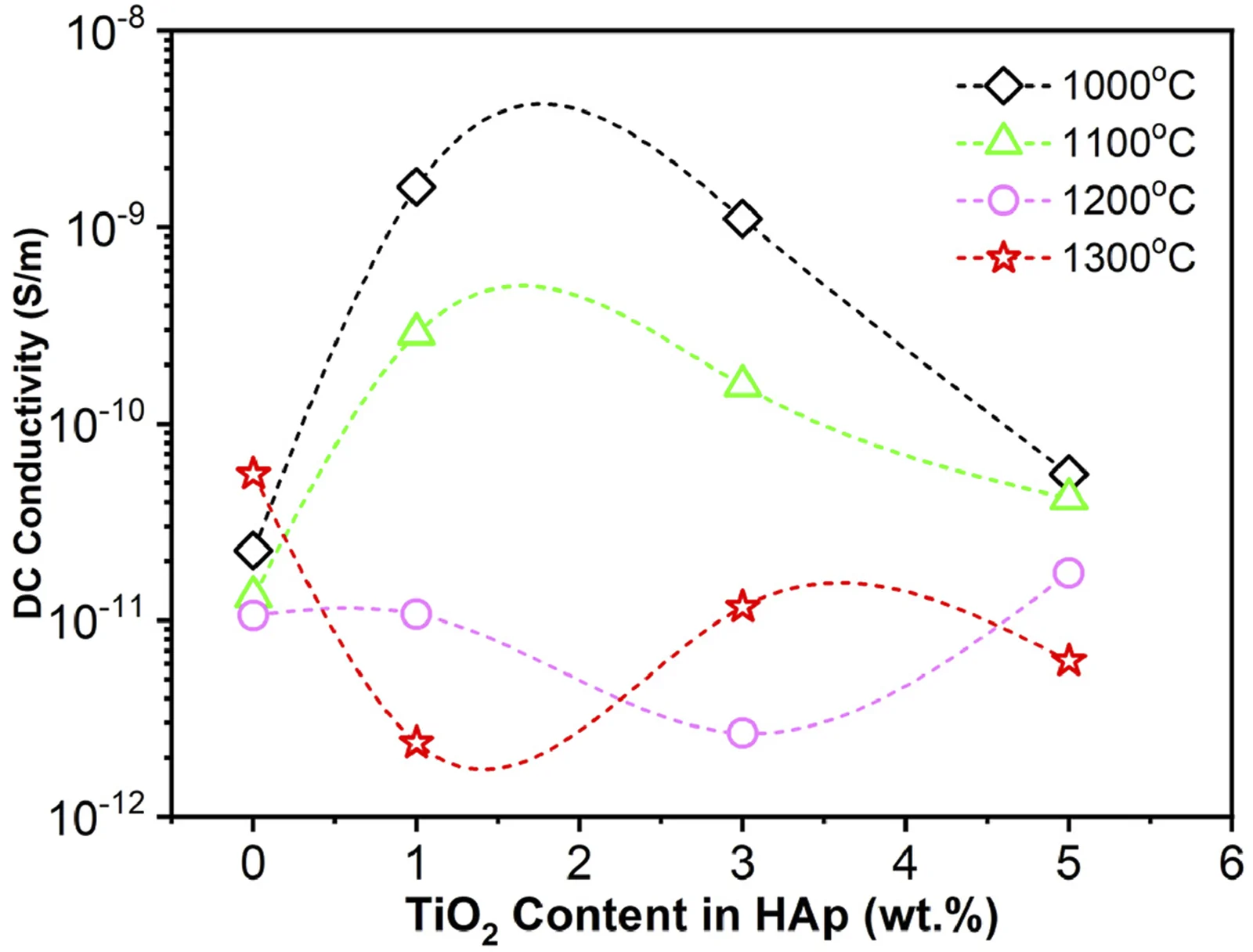
Variation of the DC conductivity of THAp *versus* TiO_2_ content at the indicated sintering temperatures.

The variation of DC conductivity is shown in Fig. 8 as a function of TiO_2_ content for the indicated sintering temperatures, and in Fig. S14 (in SI) as a function of sintering temperatures for 0–5 wt% TiO_2_ contents.

The DC conductivity of the HAp–TiO_2_ composites exhibits a pronounced non-monotonic dependence on both TiO_2_ content and sintering temperature, indicating that electrical transport is governed by a complex interplay among defect structure, microstructural evolution, and phase composition rather than by a single parameter. For the samples sintered at 1000 and 1100 °C, the addition of 1 wt% TiO_2_ produces a substantial increase in conductivity, from approximately 2 × 10^−11^ to 1.5 × 10^−9^ S m^−1^ at 1000 °C and from approximately 1 × 10^−11^ to 3 × 10^−10^ S m^−1^ at 1100 °C. Further increasing the TiO_2_ content to 3 and 5 wt% causes the conductivity to decrease markedly. This behavior suggests the existence of an optimum low TiO_2_ concentration at which defect-assisted and interfacial transport pathways are enhanced without excessive disruption of the conductive network by secondary-phase formation and structural heterogeneity.

The PALS results provide additional insight into this non-monotonic behavior. At 1000 °C, increasing the TiO_2_ content from 0 to 5 wt% causes *τ*_2_ to increase progressively from 0.481 to 0.617 ns, while *I*_2_ increases from 10.8 to 18.7%. Thus, TiO_2_ addition increases both the characteristic open volume associated with the second lifetime component and the fraction of positrons annihilating from these defect-related states. Similar systematic increases in *I*_2_ with TiO_2_ content are observed at all sintering temperatures. However, the electrical conductivity does not follow the same trend: the conductivity reaches a maximum at only 1 wt% TiO_2_ and subsequently decreases despite the continued increase in *I*_2_. Therefore, the conductivity enhancement cannot be attributed simply to an increasing concentration of vacancy-type defects or positron trapping sites.

Instead, the maximum conductivity at 1 wt% TiO_2_ for the 1000 and 1100 °C samples suggests that a limited amount of TiO_2_ may create a favorable defect and interfacial configuration for charge transport. At this low concentration, TiO_2_-induced defects and HAp/Ti-containing interfacial regions may provide additional localized states that facilitate hopping or defect-assisted transport between neighboring sites. At higher TiO_2_ contents, however, the continuing increase in *I*_2_ indicates an increasing contribution from defect-rich and interfacial environments, whereas the simultaneous decline in conductivity suggests that these sites do not form an effective long-range transport network. Instead, increased phase heterogeneity and interfacial density may promote carrier localization or trapping and interrupt otherwise continuous conduction pathways.

The evolution with sintering temperature provides further evidence that the electrical response cannot be interpreted solely in terms of defect concentration. For the 1 wt% TiO_2_ sample, conductivity decreases by nearly three orders of magnitude between 1000 and 1300 °C, while *I*_2_ increases systematically from 12.6 to 17.2%. Similarly, for the 3 wt% sample, *I*_2_ increases from 17.6 to 22.0%, although conductivity decreases strongly between 1000 and 1200 °C before partially recovering at 1300 °C. The 5 wt% sample likewise exhibits increasing *I*_2_, from 18.7 to 23.0%, without a corresponding increase in conductivity. These observations demonstrate that a higher probability of positron trapping does not necessarily imply enhanced electrical transport.

The temperature dependence of *τ*_2_ is particularly informative for the TiO_2_-containing compositions. For 3 wt% TiO_2_, *τ*_2_ decreases from 0.576 ns at 1000 °C to 0.532 ns at 1300 °C, while for 5 wt% TiO_2_ it decreases more substantially from 0.617 to 0.526 ns. At the same time, *I*_2_ increases continuously. This combination of decreasing *τ*_2_ and increasing *I*_2_ suggests that high-temperature sintering transforms the defect structure toward a larger population of comparatively smaller or more localized positron-trapping environments. Such evolution is consistent with densification and the redistribution of open-volume defects toward grain boundaries, interphase boundaries, and localized vacancy-type configurations. Importantly, however, *τ*_2_ does not decrease monotonically with temperature for every composition; therefore, its variation should be interpreted in conjunction with phase evolution and microstructural changes rather than as a direct measure of densification alone.

The behavior of the undoped HAp sample also illustrates the complexity of the relationship between PALS parameters and electrical conductivity. Its conductivity decreases slightly from 1000 to 1200 °C but increases considerably at 1300 °C, whereas *τ*_2_ changes non-monotonically from 0.481 to 0.474, 0.444, and finally 0.477 ns. The conductivity recovery at 1300 °C therefore appears more likely to be associated with changes in phase composition, grain connectivity, and intergranular transport than with a simple increase or decrease in the concentration of open-volume defects.

Taken together, the electrical and PALS results indicate that the number of positron-trapping sites alone does not determine DC conductivity. The systematic increase in *I*_2_ with TiO_2_ content and sintering temperature reflects an increasing fraction of positrons trapped in vacancy-type defects and/or interfacial environments, but these trapping sites are not necessarily electrically active or mutually connected. In particular, the pronounced conductivity maximum at 1 wt% TiO_2_ at 1000 and 1100 °C suggests that charge transport is optimized at an intermediate defect/interfacial state, whereas further TiO_2_ addition produces increasingly heterogeneous and defect-rich multiphase structures that favor carrier localization rather than long-range conduction.

Accordingly, the combined XRD, SEM, PALS, and conductivity results support a picture in which electrical transport is controlled by the nature, size, spatial distribution, and connectivity of defects and interfaces, rather than simply by their abundance. At low TiO_2_ concentration and relatively low sintering temperatures, suitable defect states and interfaces may assist hopping transport. At higher TiO_2_ contents or after extensive high-temperature phase evolution, the increasing population of localized trapping environments and multiphase boundaries can impede long-range carrier motion. PALS therefore provides complementary evidence for the evolution of the defect landscape, but the correlation with conductivity should be regarded as qualitative and indirect, since PALS probes open-volume positron-trapping states rather than electrically active charge carriers themselves.

### 3.6 AC conductivity studies

The AC electrical response of the pellets was investigated as a function of frequency. AC conductivity measurements were carried out under vacuum at room temperature (25 °C) in the frequency range of 40 Hz–10^4^ Hz. Due to instrumental limitations, conductivity values below 10 kHz were not included in the discussion or graphical representation.

**Fig. 9:**
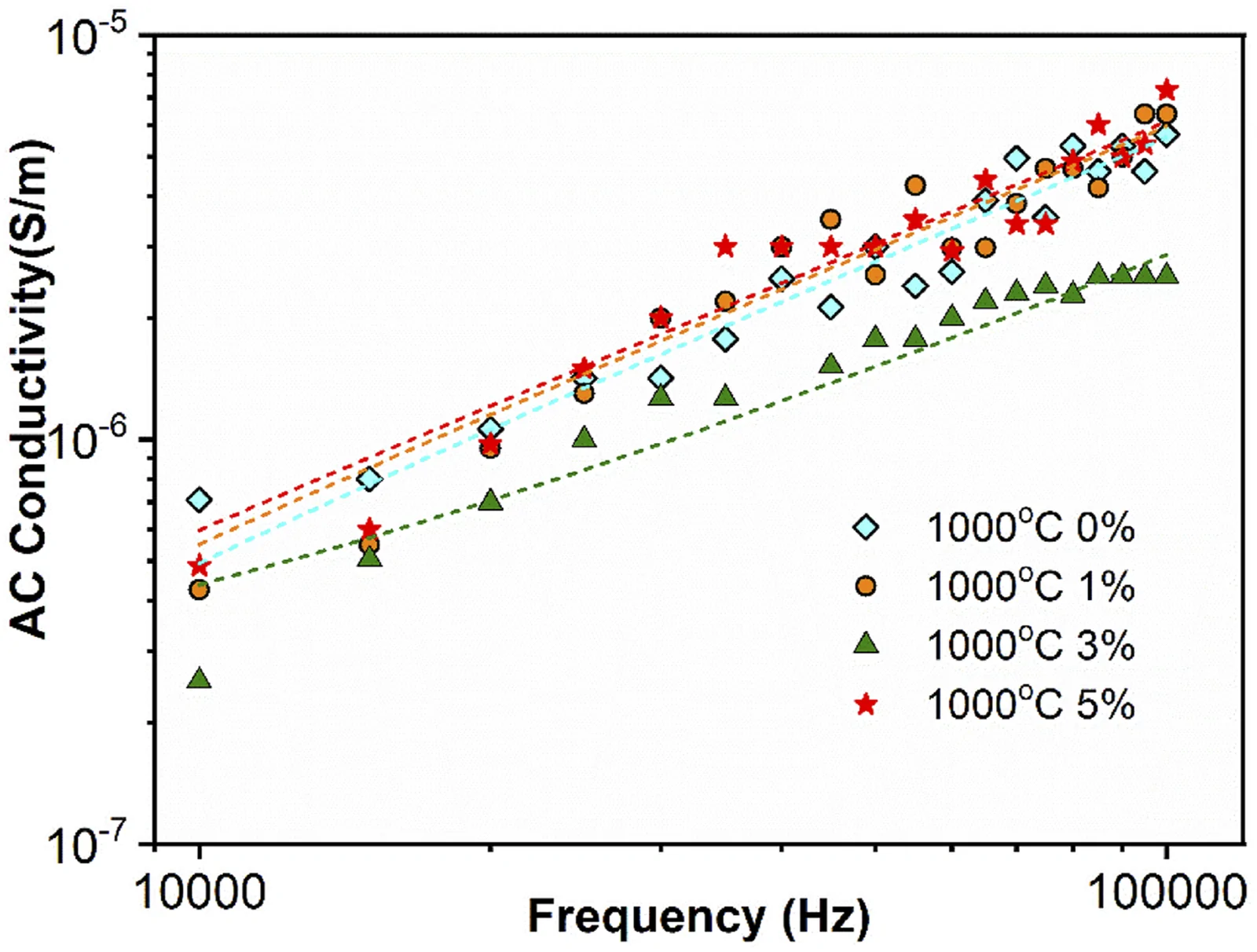
The frequency dependence of the AC conductivity for the pellets sintered at 1000 °C with the indicated doping concentrations of TiO_2_ in a vacuum at 25 °C.

Fig. 9 shows the variation of AC conductivity (*σ*_ac_) with frequency for pellets sintered at 1000 °C containing different TiO_2_ concentrations. Similar behavior was observed for the samples sintered at other temperatures. The AC conductivity values measured at 100 kHz for undoped HAp and HAp doped with 1, 3, and 5 wt% TiO_2_ were calculated as 5.67 × 10^−6^, 6.38 × 10^−6^, 2.53 × 10^−6^, and 7.32 × 10^−6^ S m^−1^, respectively.

The observed frequency-dependent increase in AC conductivity is qualitatively consistent with the universal dielectric response commonly reported for hydroxyapatite and related calcium phosphate ceramics, in which localized charge motion, polarization processes, and microstructural heterogeneity contribute to the electrical response.^24,25,27^ The AC conductivity increases gradually with frequency in the range of 10–100 kHz, exhibiting an approximately logarithmic dependence in the investigated frequency range. This behavior is characteristic of hopping-type conduction in defect-controlled ceramic systems and can be described by Jonscher's universal power law.^40–44^

The conductivity spectra were fitted using Jonscher's power law, *σ*_ac_(*ω*)≡*aω*^*n*^, with the fitting parameters given in Table S3 for 0 and 5 wt% TiO_2_ (see SI). For most compositions and sintering temperatures, the exponent *n* lies between 0.82 and 0.97, consistent with a sublinear dispersive response associated with localized hopping and polarization processes. Slightly superlinear values were obtained for undoped HAp sintered at 1100 °C and the 5 wt% TiO_2_ sample sintered at 1000 °C.

The increase in conductivity with frequency suggests that charge transport is dominated by localized hopping between defect states and interfacial trapping centers rather than by long-range free-carrier conduction.

The frequency dependence of AC conductivity remains relatively similar for all TiO_2_ concentrations, indicating that TiO_2_ addition in the range of 1–5 wt% does not significantly alter the dominant AC conduction mechanism. This behavior differs from the DC conductivity response, which is strongly influenced by defect connectivity and interfacial trapping effects.

**Fig. 10:**
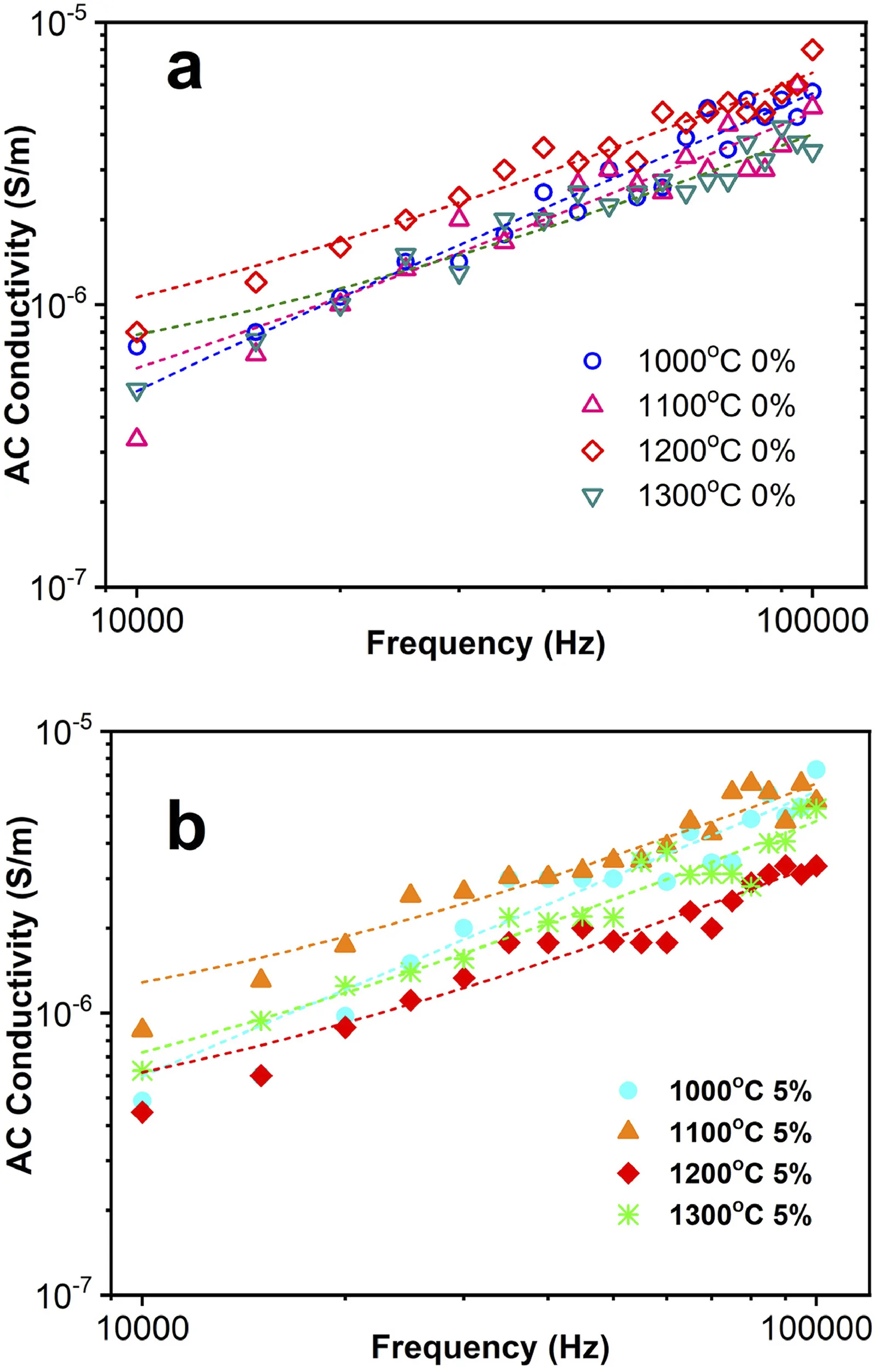
Frequency dependence of AC conductivity for HAp pellets (a) undoped and (b) doped with 5 wt% TiO_2_ and sintered at the indicated temperatures in a vacuum (10^−2^ mbar) at 25 °C.

To evaluate the influence of sintering temperature on the AC electrical response of THAp composites, the variation of AC conductivity with frequency was investigated for different compositions and temperatures. Fig. 10a shows the AC conductivity behavior of undoped HAp pellets sintered at temperatures between 1000 and 1300 °C, while Fig. 10b shows the corresponding results for pellets containing 5 wt% TiO_2_. Similar trends were also observed for samples containing 1 and 3 wt% TiO_2_.

The undoped HAp pellets sintered at 1000, 1100, 1200, and 1300 °C exhibited AC conductivity values at 100 kHz of 5.67 × 10^−6^, 5.00 × 10^−6^, 8.00 × 10^−6^, and 3.50 × 10^−6^ S m^−1^, respectively. For the pellets containing 5 wt% TiO_2_, the corresponding values were 7.32 × 10^−6^, 5.65 × 10^−6^, 3.33 × 10^−6^, and 5.31 × 10^−6^ S m^−1^. Although some variation with sintering temperature is observed, the overall AC response remains comparatively stable relative to the DC conductivity behavior. This indicates that AC conduction is less sensitive to long-range defect connectivity and is governed mainly by localized polarization and short-range hopping processes.

The observed AC conductivity behavior is closely related to the defect evolution identified by PALS. In particular, the increase in the defect-related intensity component (*I*_2_) with TiO_2_ addition and sintering temperature indicates a higher fraction of positron trapping at vacancy-type defects, grain boundaries, and HAp/Ti-containing interfacial regions. These defect-rich regions can act as localized hopping and polarization centers under an alternating electric field. However, the corresponding decrease in the lifetime component (*τ*_2_) at higher sintering temperatures suggests that the open-volume defects become smaller and more localized as densification proceeds. Consequently, although the number of trapping sites increases, their spatial connectivity and effective hopping distance decrease.

Unlike DC conductivity, which requires continuous conduction pathways across the material, AC transport can be sustained through localized hopping between nearby defect states. Therefore, the AC response does not increase proportionally with *I*_2_, despite the increase in defect-related trapping sites. The combined AC conductivity and PALS results indicate that AC transport in THAp composites is governed primarily by localized defect polarization and short-range hopping mechanisms, whereas DC transport is more strongly controlled by the connectivity of defect networks and interfacial conduction pathways. These findings highlight the important role of defect structure and interfacial heterogeneity in determining the electrical behavior of THAp composites.

## 4 Conclusions

The incorporation of TiO_2_ into HAp leads to significant phase evolution and defect restructuring with increasing sintering temperature. XRD results indicate that above 1100 °C, HAp progressively decomposes into β-TCP and reacts with TiO_2_ to form secondary phases such as CaTiO_3_. These transformations introduce chemical heterogeneity and generate interfacial regions within the composite structure. SEM observations further confirm a concurrent microstructural evolution from a porous structure at 1000 °C to a dense and relatively uniform structure at 1200 °C, followed by pronounced grain coarsening at 1300 °C. From a microstructural perspective, 1200 °C provides the most balanced condition in terms of densification and controlled grain growth.

PALS results reveal a systematic increase in the fraction of positrons trapped at defect-related and interfacial environments with increasing TiO_2_ content and sintering temperature, as evidenced by the continuous increase in *I*_2_, while the composition- and temperature-dependent evolution of *τ*_2_ reflects changes in the characteristic size and nature of these open-volume trapping sites. For the higher TiO_2_ contents, the general decrease in *τ*_2_ with increasing sintering temperature, accompanied by increasing *I*_2_, suggests a redistribution of positron trapping toward a larger population of comparatively smaller and more localized defect or interfacial environments. These changes are consistent with the densification, secondary-phase formation, and interfacial heterogeneity revealed by XRD and SEM. The non-monotonic DC conductivity behavior indicates that charge transport is governed not simply by defect abundance but by the combined effects of defect character, phase composition, microstructure, and pathway connectivity. In particular, the conductivity enhancement at 1 wt% TiO_2_ for samples sintered at 1000 and 1100 °C suggests a favorable defect/interfacial configuration for charge transport, whereas further TiO_2_ addition generally promotes structural heterogeneity and localized trapping environments that hinder long-range carrier transport. In contrast, the comparatively less variable AC conductivity response is consistent with localized hopping and polarization processes. Overall, the combined XRD, SEM, PALS, and electrical measurements demonstrate that phase evolution, microstructural development, and interfacial defect structures collectively govern the structural and electrical properties of the HAp–TiO_2_-derived calcium phosphate–titanate composites.

Although the present study establishes clear correlations between phase evolution, defect structure, and electrical behavior, further investigations employing quantitative Rietveld refinement, quantitative grain-size analysis, EDS elemental mapping, XPS, Raman spectroscopy, TEM, impedance spectroscopy, and temperature-dependent conductivity measurements would provide a more comprehensive understanding of phase composition, microstructural evolution, the spatial distribution of Ti-containing phases, grain and grain-boundary contributions, and the activation mechanisms governing charge transport in THAp composites. Such complementary analyses will further elucidate the interplay between microstructure, defect evolution, and electrical transport, enabling more effective defect engineering of calcium phosphate–titanate ceramics.

## Conflicts of interest

There are no conflicts to declare.

## Supplementary Material

RA-OLF-D6RA04627D-s001

## Data Availability

The data supporting this article have been included as part of the supplementary information (SI). Supplementary Information: particle-size analysis, additional SEM/BEI and EDS results, PALS data, hardness and density data, and Jonscher power-law fitting parameters. See DOI: https://doi.org/10.1039/d6ra04627d.
