# Supplementary material for: Defect evolution and electrical response in HAp–TiO2-derived calcium phosphate–titanate composites investigated by positron annihilation lifetime spectroscopy
Source: RSC Adv. 2026 Aug 24;16(42):50150–62. doi: 10.1039/d6ra04627d (PMC13502244; doi:10.1039/d6ra04627d)
Supplement: Supplementary file 1 [file RA-016-D6RA04627D-s001.pdf]

Supplementary Information (SI) for RSC Advances.
This journal is © The Royal Society of Chemistry 2026

SUPPLEMENTARY INFORMATION FOR

Defect Evolution and Electrical Response in HAp–TiO_2-Derived Calcium Phosphate–Titanate Composites Investigated by Positron Annihilation Lifetime Spectroscopy

Uğur Yahşi[1*], Şeyma Şimal Ökmen[1], Fatih Dumludağ[1], Cumali Tav[1], Ziya Engin Erkmen[2*]

[1]Physics Department, Faculty of Science, Marmara University, 34722 Kadikoy, Istanbul, Türkiye

[2]Metallurgical and Materials Engineering Department, Faculty of Engineering, Marmara University, 34854 Maltepe, Istanbul, Türkiye

S1. The Particle Size Analysis

The particle size analysis was performed with a particle/zeta sizer (Malvern Pananalytical, Zetasizer ver. 8.02). Figure S1 (*a*) and (*b*) shows all the detailed results of the particle size measurements of commercial HA and titania, respectively, measured at 25 °C.

System

**Temperature (°C):**	25.0	**Duration Used (s):**	50
**Count Rate (kcps):**	153.6	**Measurement Position (mm):**	4.65
**Cell Description:**	Clear disposable zeta cell	**Attenuator:**	9

Results

			Size (d.n...	% Intensity:	St Dev (d.n...
**Z-Average (d.nm):**	945.4	**Peak 1:**	697.8	100.0	102.4
**PdI:**	1.000	**Peak 2:**	0.000	0.0	0.000
**Intercept:**	0.856	**Peak 3:**	0.000	0.0	0.000

Result **Refer to quality report**

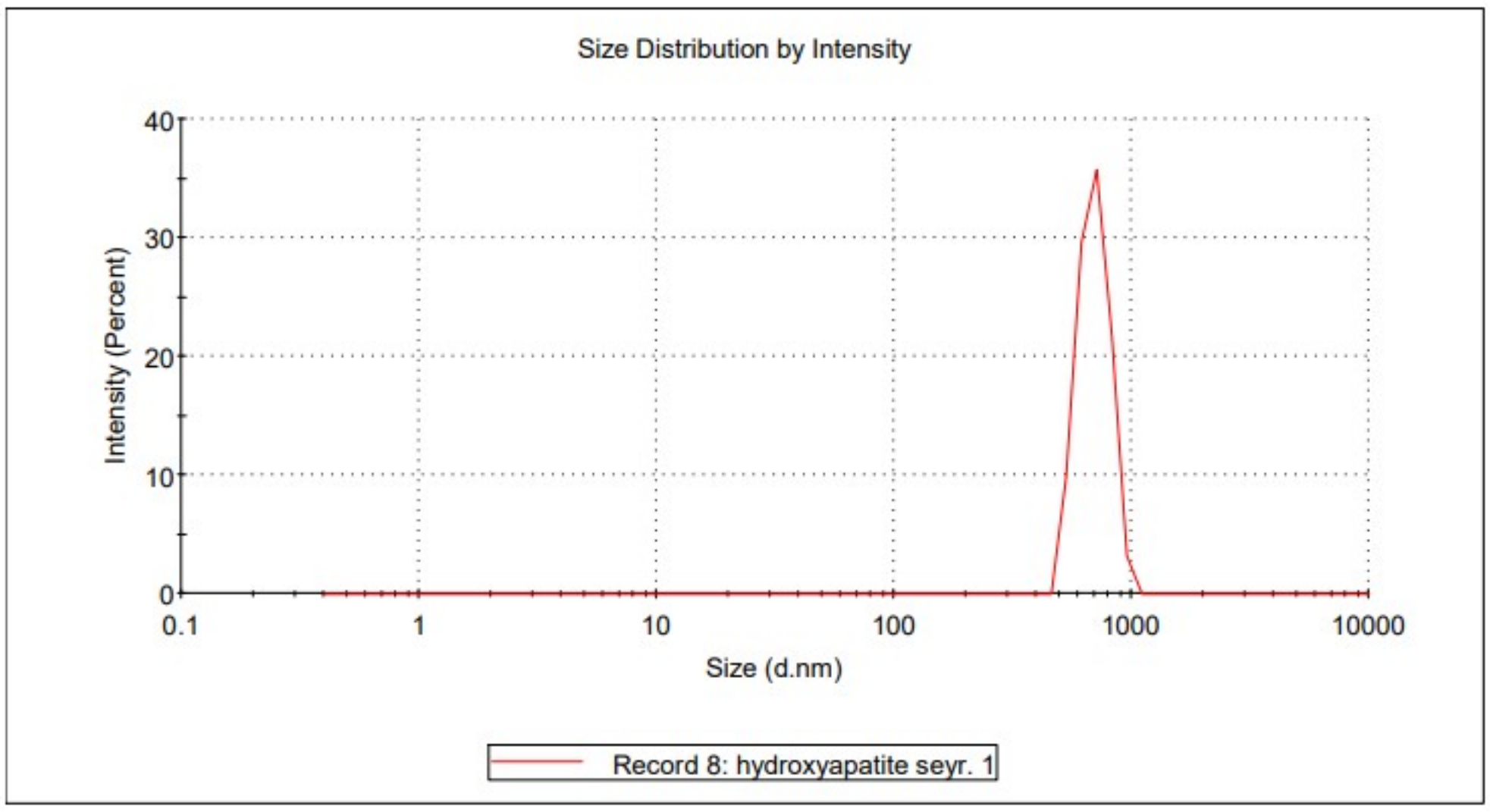

Figure S1. The results of the particle size measurement of commercial HA

System

Temperature (°C):	25.0	Duration Used (s):	50
Count Rate (kcps):	144.0	Measurement Position (mm):	4.65
Cell Description:	Clear disposable zeta cell	Attenuator:	6

Results

			Size (d.n...	% Intensity:	St Dev (d.n...
Z-Average (d.nm):	760.6	Peak 1:	634.2	100.0	103.3
PdI:	1.000	Peak 2:	0.000	0.0	0.000
Intercept:	0.767	Peak 3:	0.000	0.0	0.000

Result Refer to quality report

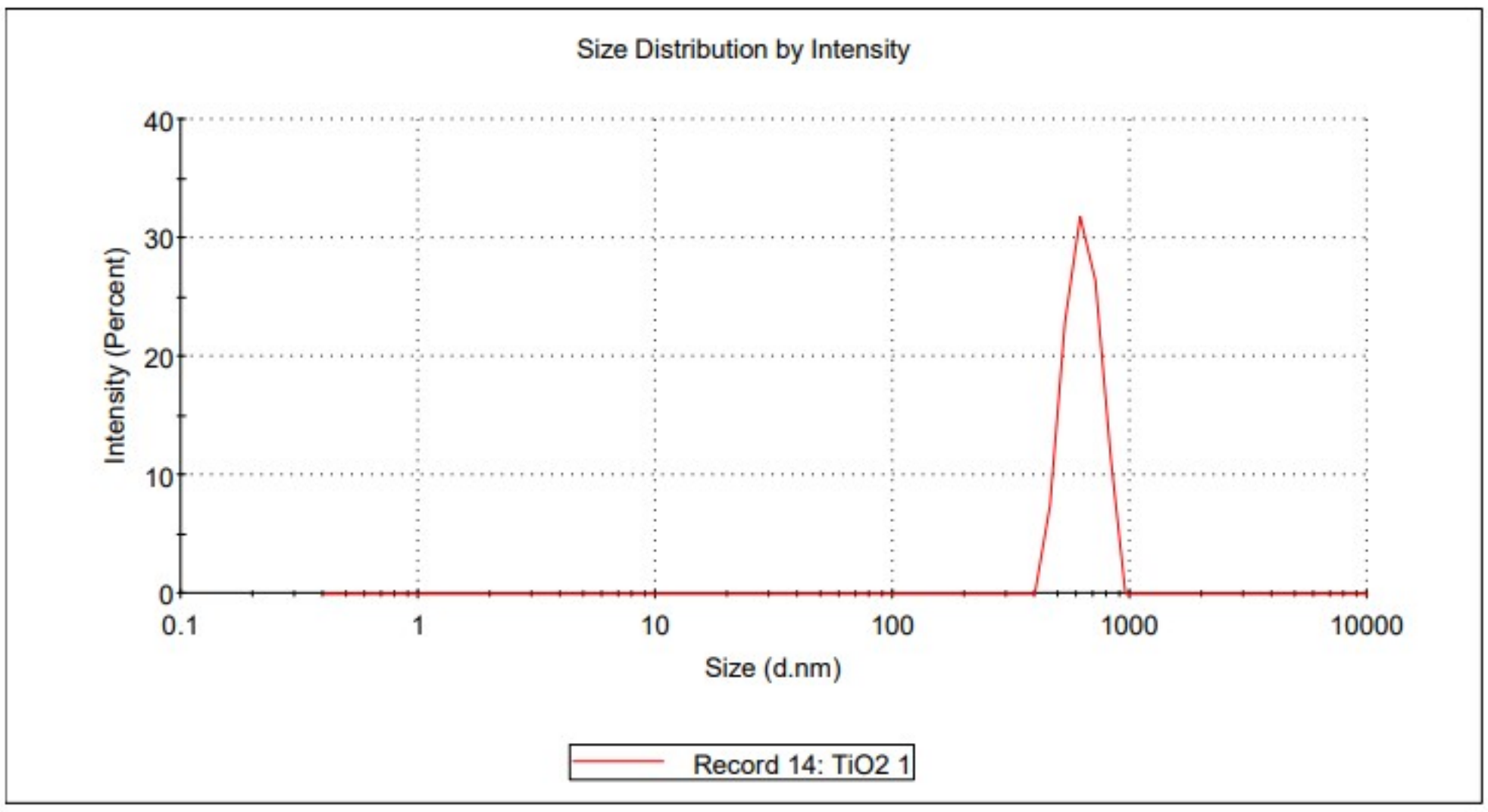

Figure S2. The results of the particle size measurement of commercial titania

S2. Scanning Electron Microscopy (SEM) and Energy Dispersive Spectroscopy (EDS)

SEM analysis

SEM analysis was performed using a Zeiss SEM EVO MA10 and samples were coated by gold using a Quorum SC7620 PVD apparatus. The HAp samples containing 5wt% TiO_2 as additive sintered at 1000, 1100, 1200, 1300 °C were used in order to observe the temperature effect as variable.

Figure S3 illustrates the HAp sample with 5wt% titania sintered at 1000 °C for 1 hour at (*a*) 5Kx, (*b*) 10Kx, and (*c*) 10Kx at BEI mode. The white particulates seen in BEI are dispersed titania on the sample surface. In Fig. S3(*c*) particulates of titania are visible in BEI mode whereas they are merged with HAp matrix in Fig. S3(*d*).

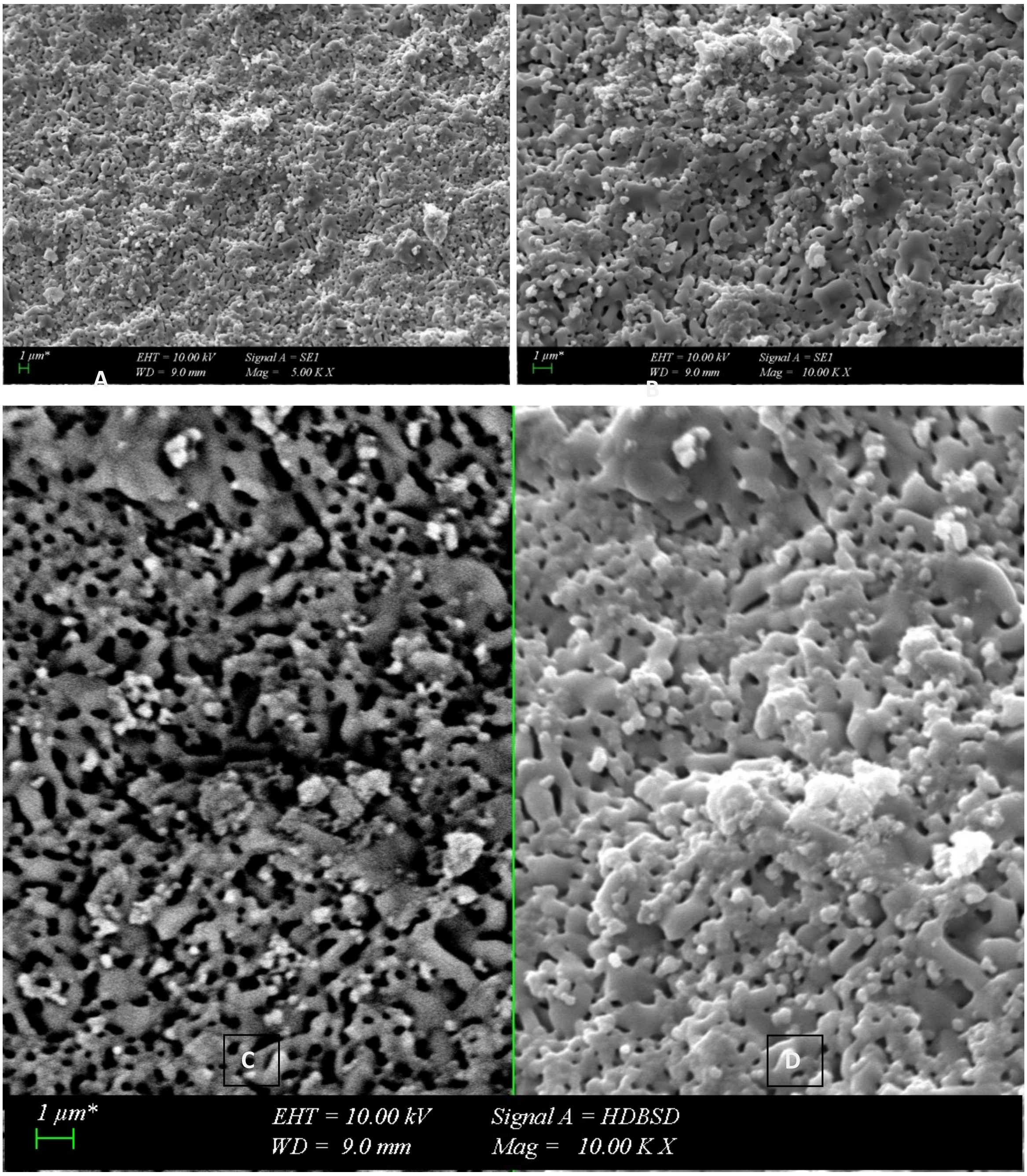

Figure S3. SEM image taken at (*a*) 5Kx, (*b*) 10Kx, and (*c*) at BEI mode for 1 hour for the samples with 5wt% titania sintered at 1000 °C.

Figure S4 indicates HAp sample with 5wt% titania sintered at 1100 °C for 1 hour with the same order of magnification as in Fig. S3. Comparing with Fig. S3, grains are grown and lesser porosity are present.

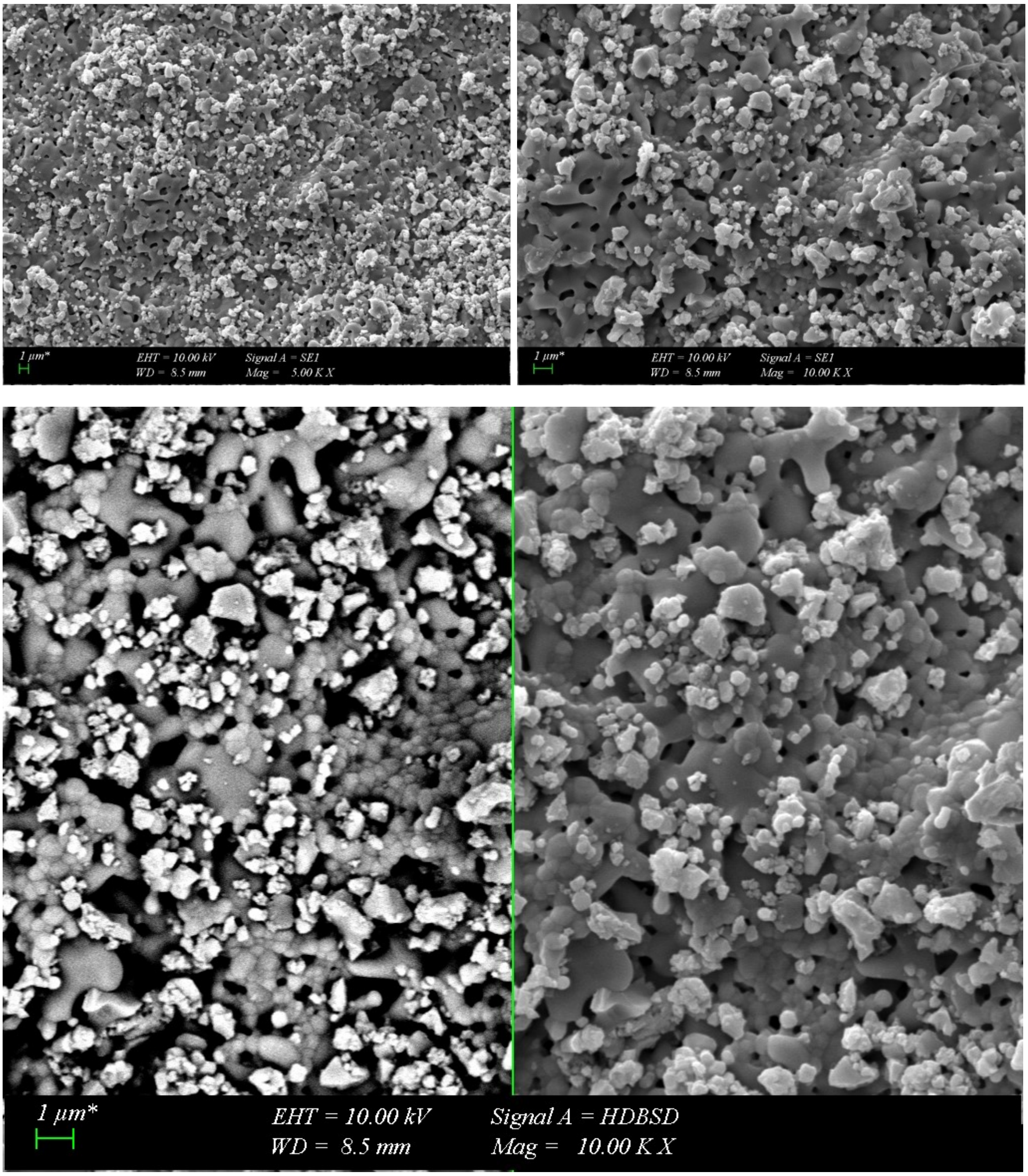

Figure S4. SEM image taken at (*a*) 5Kx, (*b*) 10Kx, and (*c*) at BEI mode for 1 hour for the samples with 5wt% titania sintered at 1100 °C.

Figure S4 indicates HAp samples with 5w% titania sintered at 1200 °C for 1 hour. Comparing with Figs. S3-S4 grains are sintered better with almost complete necking and few porosities are present.

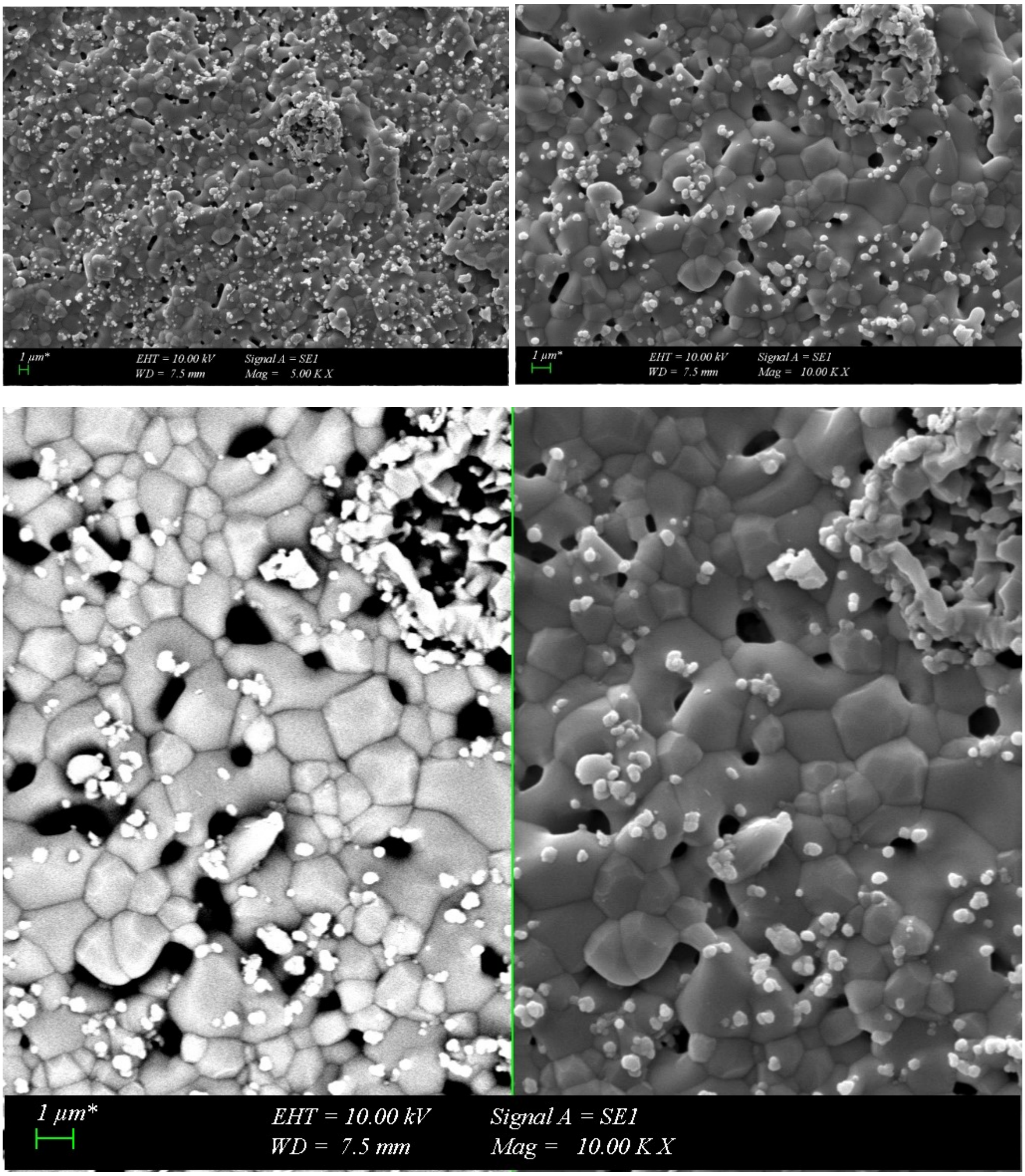

Figure S5. SEM image taken at (*a*) 5Kx, (*b*) 10Kx, and (*c*) 10Kx at BEI mode for 1 hour for the samples with 5wt% titania sintered at 1200 °C.

Figure S6 indicates HAp samples with 5w% titania sintered at 1300 °C for 1 hour. Grains are sintered almost completely and titania particulates are embedded partly into HAp matrix.

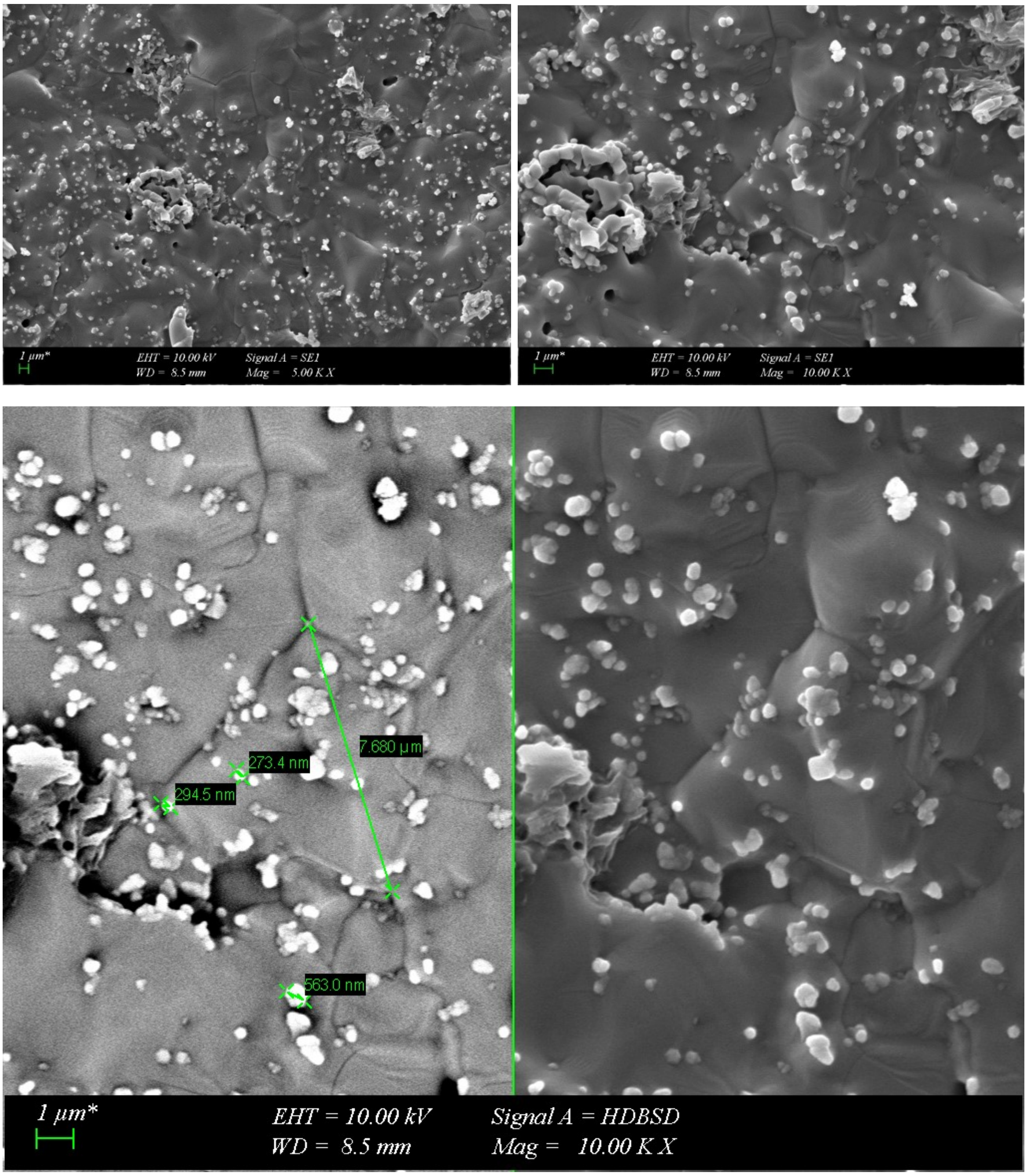

Figure S6. SEM image taken at (*a*) 5Kx, (*b*) 10Kx, and (*c*) 10Kx at BEI mode for 1 hour for the samples with 5wt% titania sintered at 1300 °C.

EDS Analysis

EDS analysis was performed on selected matrix and particulate regions of the sample sintered at 1300 °C. The spectra confirm the presence of Ca, P, O, and Ti, indicating the coexistence of calcium phosphate and Ti-containing regions in the sintered composite. However, EDS provides only semi-quantitative elemental information and cannot distinguish between TiO_2, calcium titanate phases, or Ti species incorporated into calcium phosphate phases. Therefore, EDS results are used only for elemental confirmation, while phase identification is based on XRD and defect characterization is based on PALS.

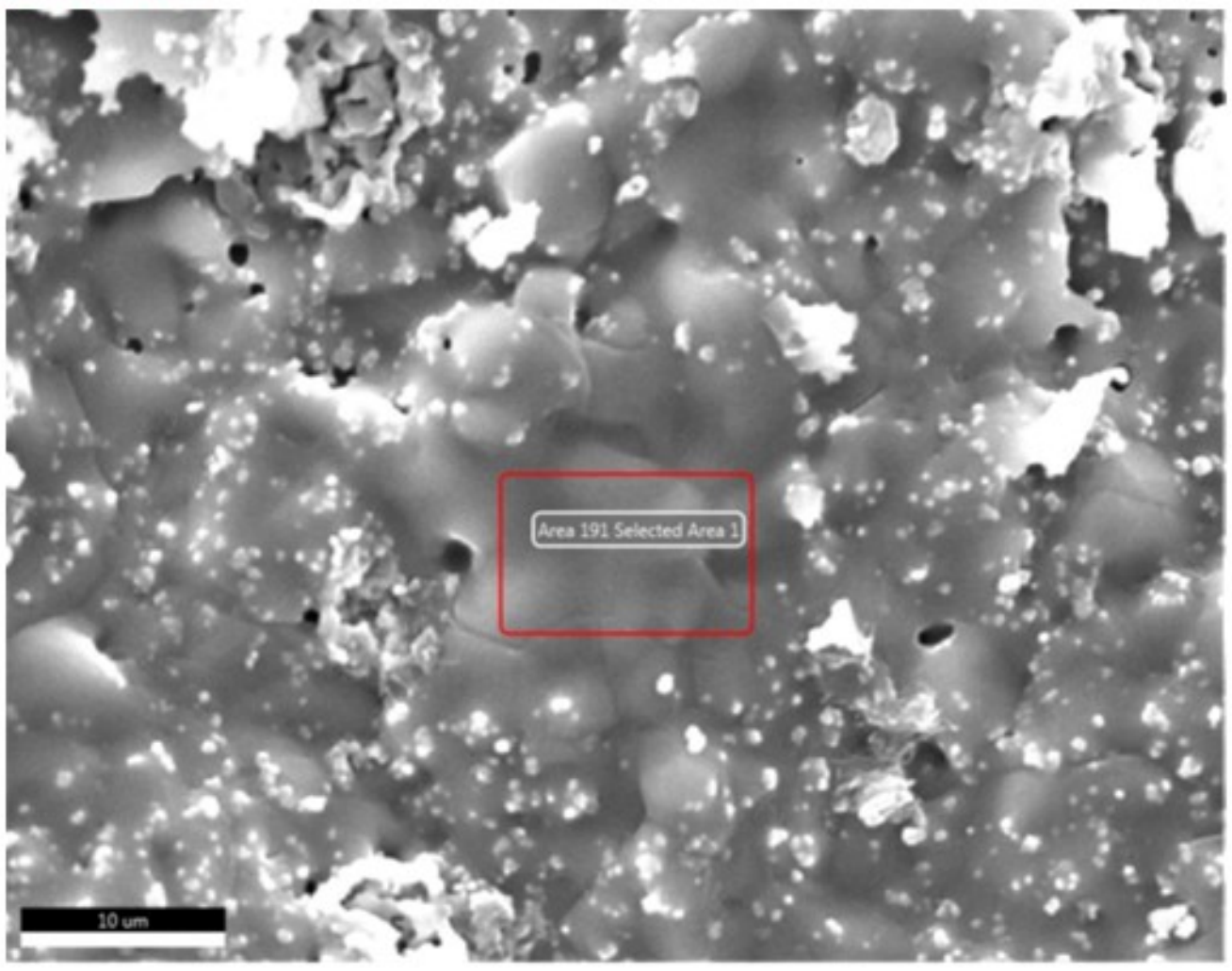

Smart Quant Results

Element	Weight %	Atomic %	Error %
O K	34.7	54.91	12.12
P K	21.08	17.23	3.57
CaK	43.52	27.49	2.31
TiK	0.71	0.37	19.9

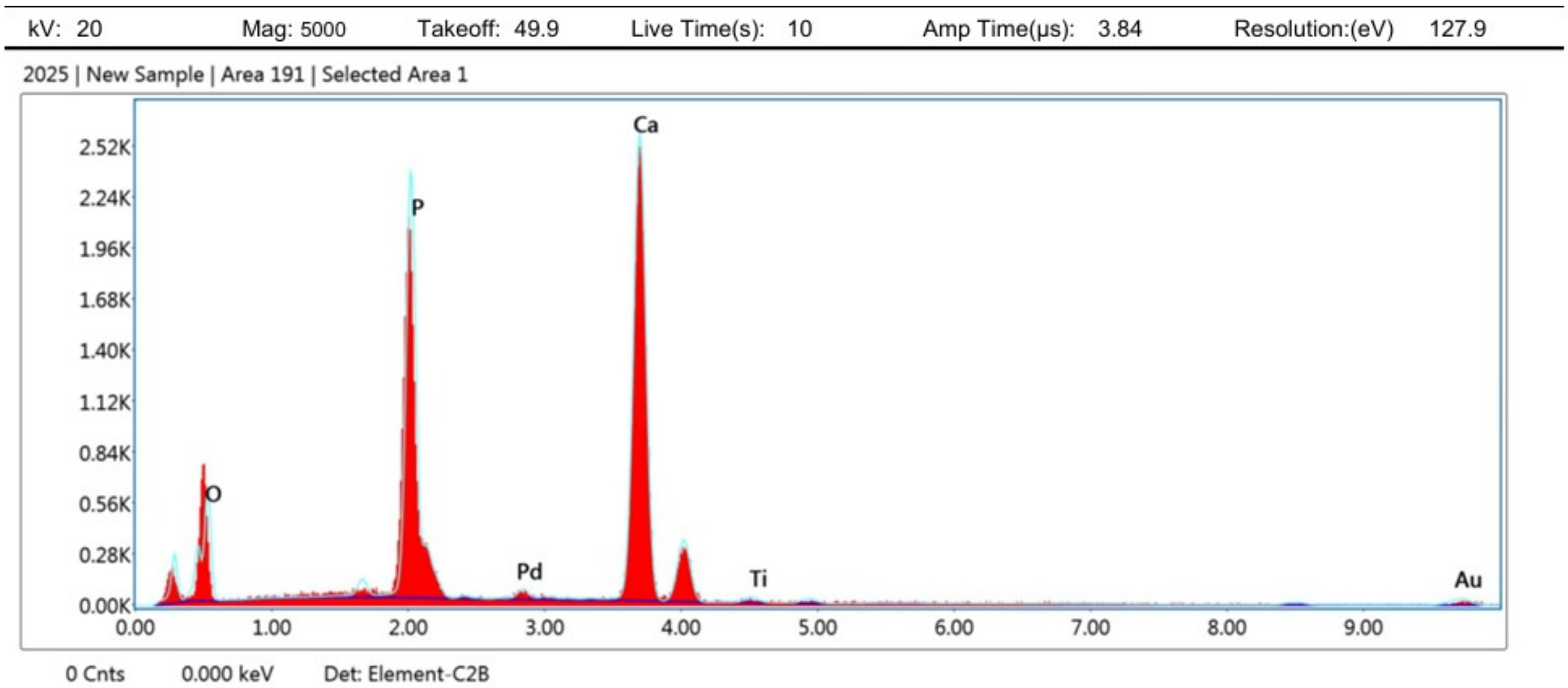

Figure S7. EDS analyses result for the selected matrix area in red rectangle

As seen the matrix exempt from the titania particulates contains the HAp constitutive elements. In contrary, the elemental analysis performed on the merged particulate reveals also the presence of Ca, P elements beside Ti and O indicating that some chemical reactions forming calcium titanate as proved additionally following XRD. A significant grain growth for HAp matrix occurred during sintering from 1000 °C to 1300 °C from nearly 1 micron up to 7.68-micron seventh order on average whereas TiO_2 seems to conserve their size (250 nm on average). Minor Au and Pd peaks originate from the sputter-coating used to improve conductivity for SEM-EDS analysis.

This can also be a proof of $CaTiO_3$ presence (formation) on the matrix: 8.55wt% Ti/39.11wt O = 0.218 fairly matches with stochiometric value: 22/48 = 0.458. Some oxygens atoms may originate from the spurrious excitation of the matrix also showing nearly 12% error measurement.

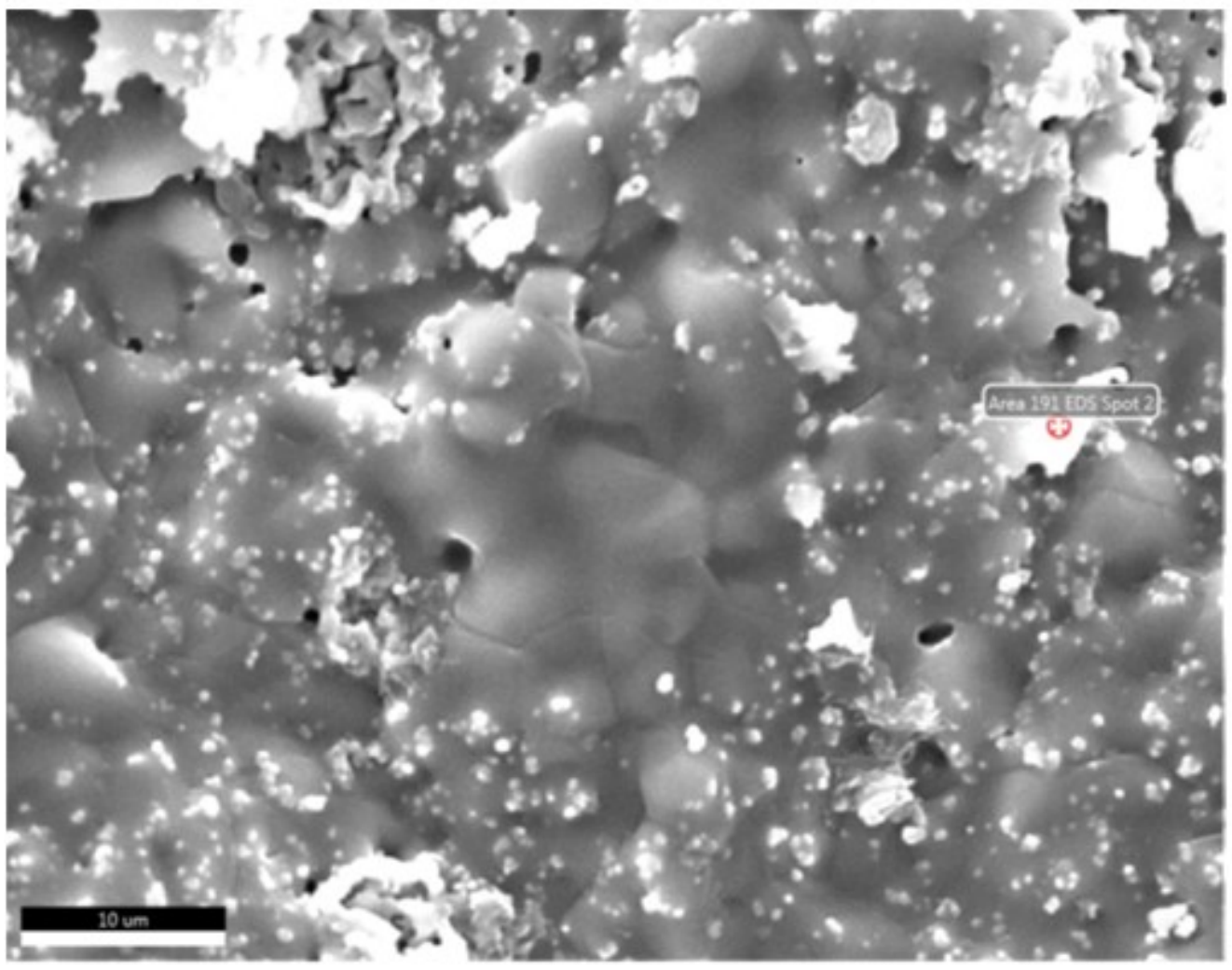

Smart Quant Results

Element	Weight %	Atomic %	Error %
O K	39.11	60.44	11.82
P K	15.82	12.63	4.03
CaK	36.51	22.52	2.36
TiK	8.55	4.41	5.04

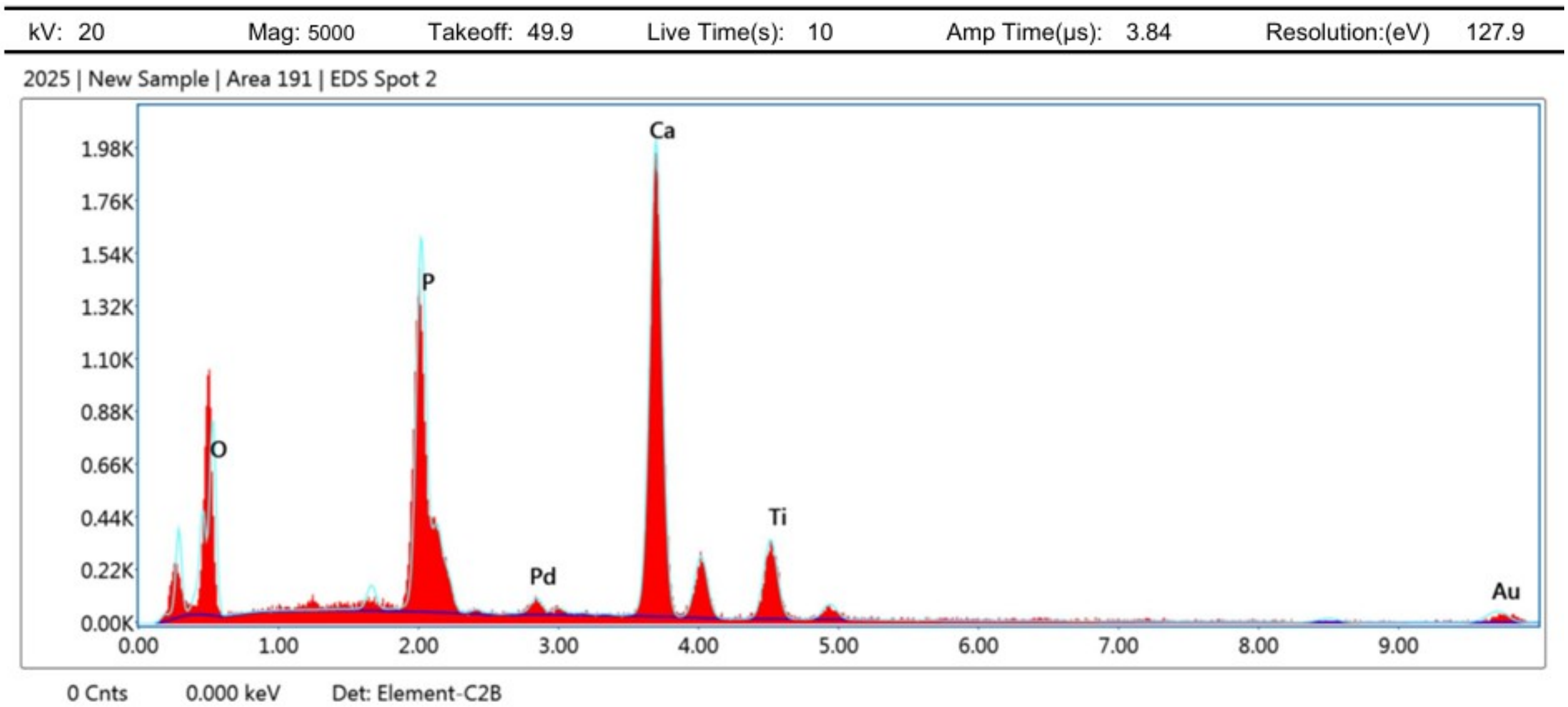

Figure S8. EDS analyses result for the selected particulate area in red circle

S3. Hardness Measurement

Microhardness measurements were taken with a force of 0.5 N applied to the diamond indenter. The means and standard deviations of at least three measurements were calculated and are presented in Table 1.

Table S1. The microhardness of the THAp composites highlights the effects of varying compositions and changes in temperature.

TiO_2 wt% \ T (°C)	1000	1100	1200	1300
0	293 ± 29	335 ± 17	552 ± 36	659 ± 40
1	67.9 ± 2.7	98.3 ± 11	367 ± 16	512 ± 48
3	69.1 ± 4.3	72.6 ± 7.8	218 ± 11	405 ± 13
5	50.4 ± 6.1	67.5 ± 6.1	103 ± 11	292 ± 28

S4. PALS results

PALS parameters as lifetimes (τ_1, τ_2 and τ_3) and intensities (I_2 and I_3) in terms of sintering temperature for various TiO_2 contents are tabulated in Table S2.

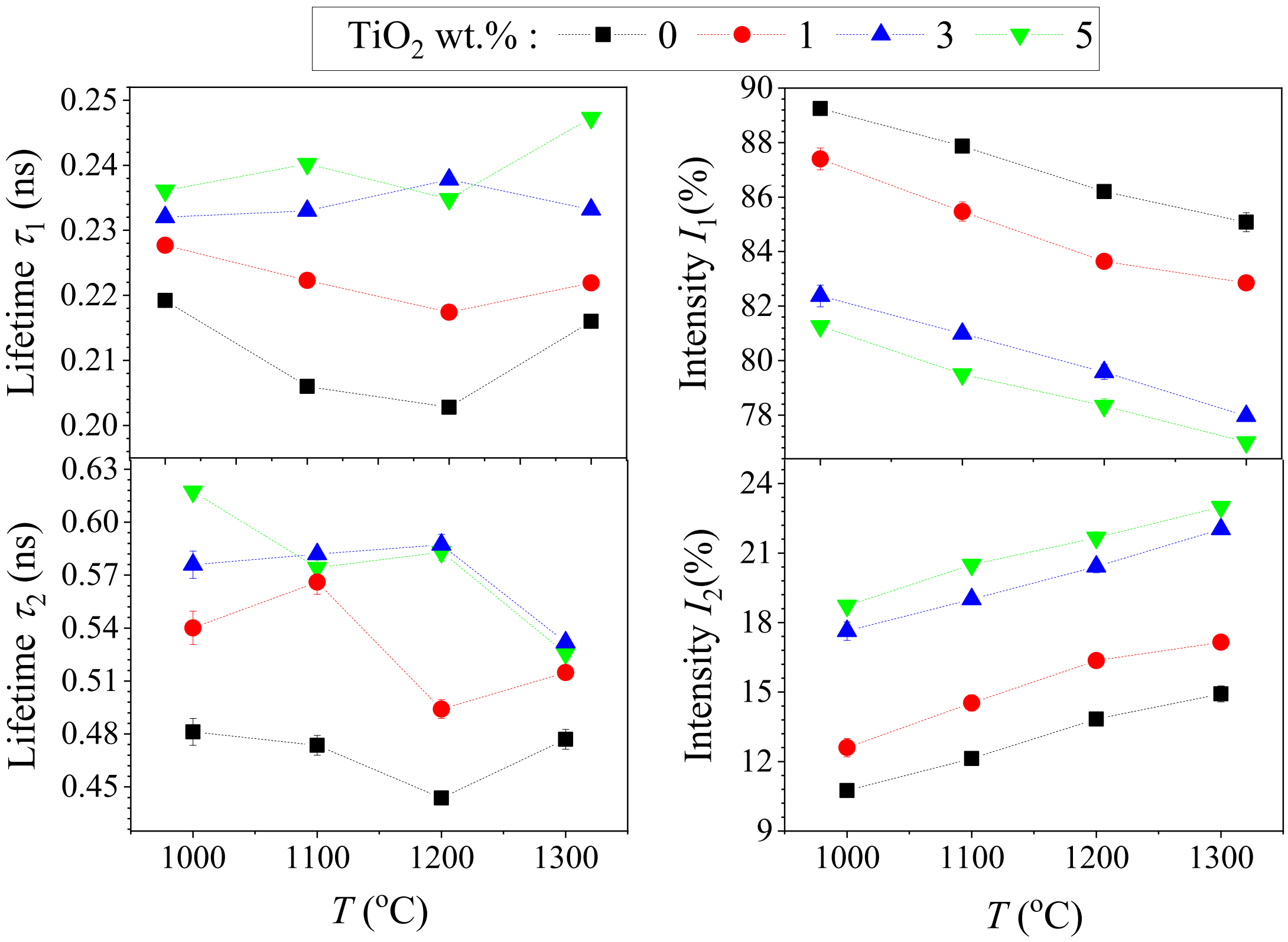

Figure S9. Positron annihilation lifetime parameters: lifetimes τ_1 and τ_2, and their intensities I_1, and I_2 versus sintering temperature.

Table S2. PALS parameters as lifetimes (τ_1 and τ_2) and intensities (I_1 and I_2) in terms of sintering temperature for various TiO_2 contents.

Sintering T(°C)	TiO_2 Content (%)	τ_1 (ns) (±0.001)	τ_2 (ns) (±0.004)	I_1 (%) (±0.4)	I_2 (%) (±0.2)
1000°C	0	0.219	0.481	89.3	10.8
	1	0.228	0.540	87.4	12.6
	3	0.232	0.576	82.4	17.6
	5	0.236	0.617	81.3	18.7
1100°C	0	0.206	0.474	87.9	12.1
	1	0.222	0.566	85.5	14.5
	3	0.233	0.582	81.0	19.0
	5	0.240	0.574	79.5	20.5
1200°C	0	0.203	0.444	86.2	13.8
	1	0.217	0.494	83.6	16.4
	3	0.238	0.587	79.6	20.4
	5	0.235	0.583	78.3	21.7
1300°C	0	0.216	0.477	85.1	14.9
	1	0.222	0.515	82.9	17.2
	3	0.233	0.532	78.0	22.0
	5	0.247	0.526	77.0	23.0

The intensities were normalized as $I_1 + I_2 = 1$.

S5. XRD Results

XRD Analysis direct outcomes are provided through Figures S11 to S14. A summary is also given in the manuscript in which only the major phases are labelled such that TiO_2 for 1300 ^{o}C sintered sample although it is also present as a minor phase at lower temperatures.

Comparing S12 with S13, it can be stated that the major phase transformation starts above 1100 °C with the dissappearance of diffraction spikes (32° < 2θ < 33°) belonging to Calcium Hydroxide phases (JSPDS: 01-080-3958, 01-074-8771)

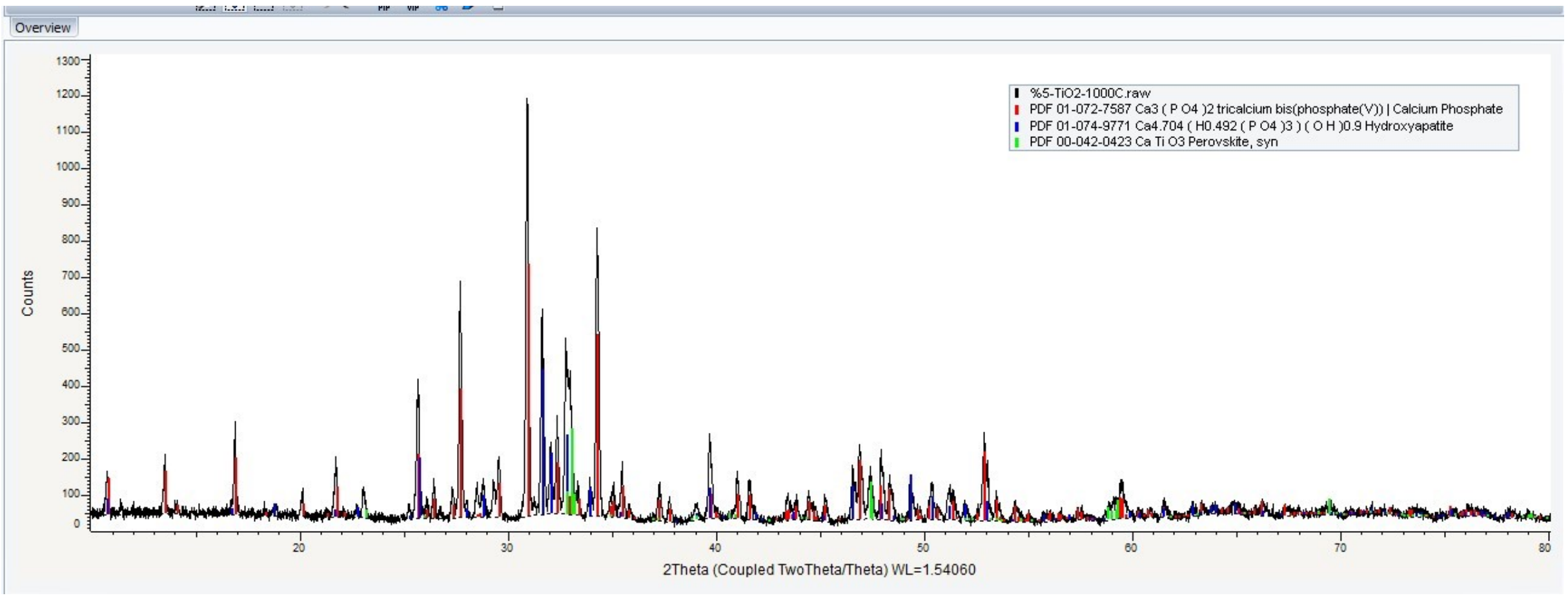

Figure S10. XRD output for 5wt% TiO_2 doped Hap sintered at 1000 ^oC for 3 hours

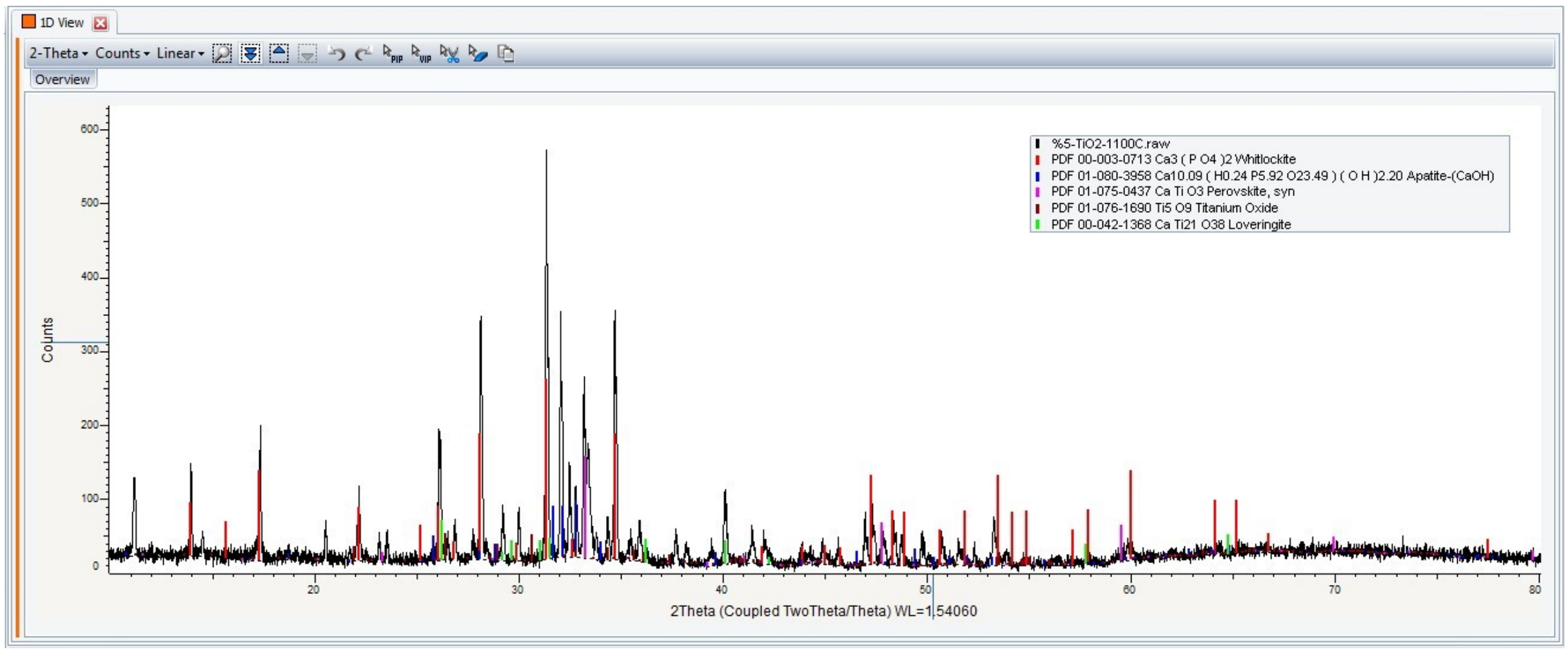

Figure S11. XRD output for 5wt% TiO_2 doped HAp sintered at 1100 ^oC for 3 hours

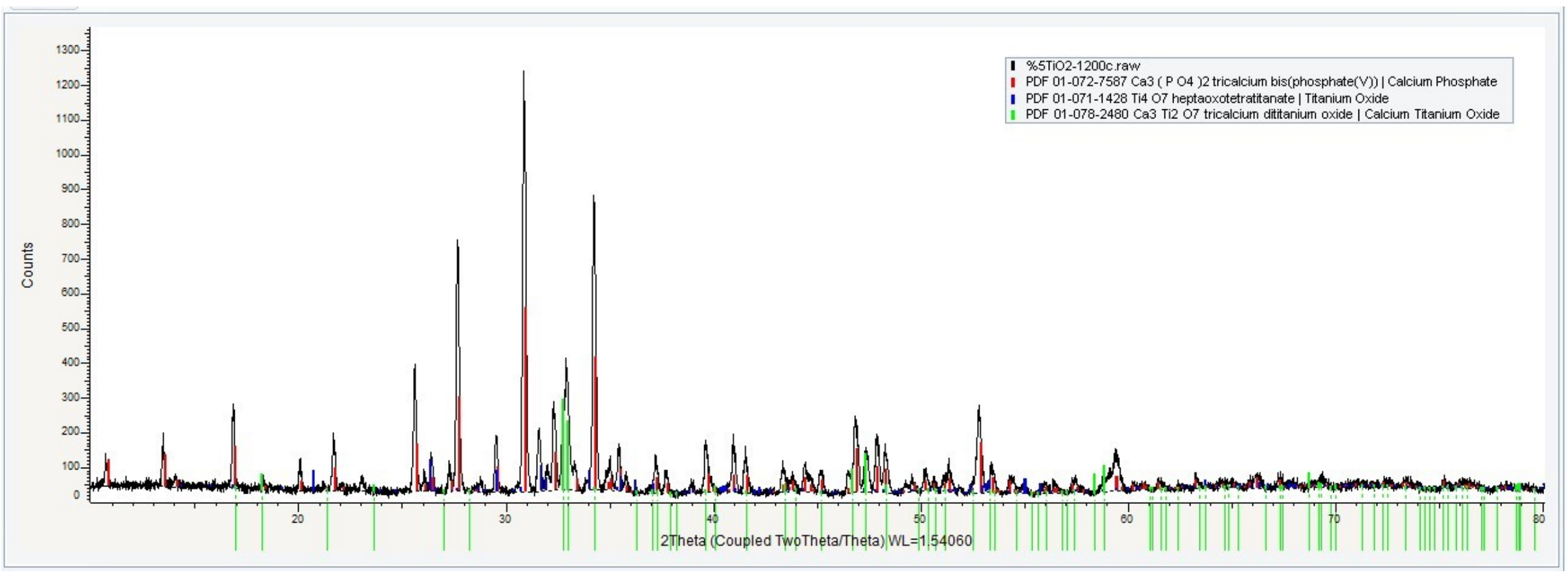

Figure S12. XRD output for 5wt% TiO_2 doped HAp sintered at 1200 ^oC for 3 hours

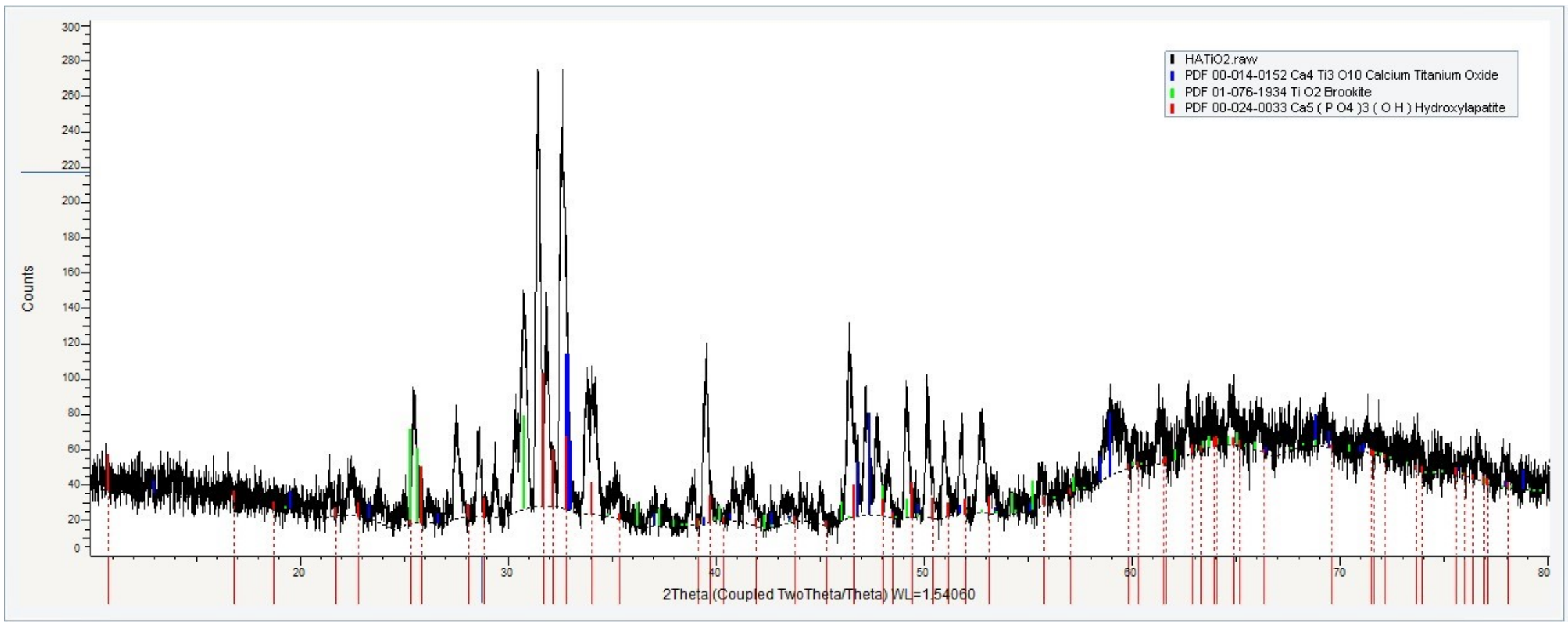

Figure S13. XRD output for 5wt% TiO_2 doped HAp sintered at 1300 ^oC for 1 hour

S6. Conductivity results

Variation of the DC conductivity of HAp/TiO_2 versus the sintering temperature for 1–5 wt.% TiO_2 are given in Table S3 and plotted in Fig. S14.

Table S3. DC conductivity values (in S/m) for the sintering temperatures of 1000-1300 °C and 0–5 wt.% TiO_2

wt% \ T(C)	1000°C	1100°C	1200°C	1300°C
**0**	2.256E-11	1.318E-11	1.057E-11	5.545E-11
**1**	1.601E-09	2.866E-10	1.072E-11	2.374E-12
**3**	1.105E-09	1.561E-10	2.653E-12	1.167E-11
**5**	5.545E-11	4.155E-11	1.738E-11	6.191E-12

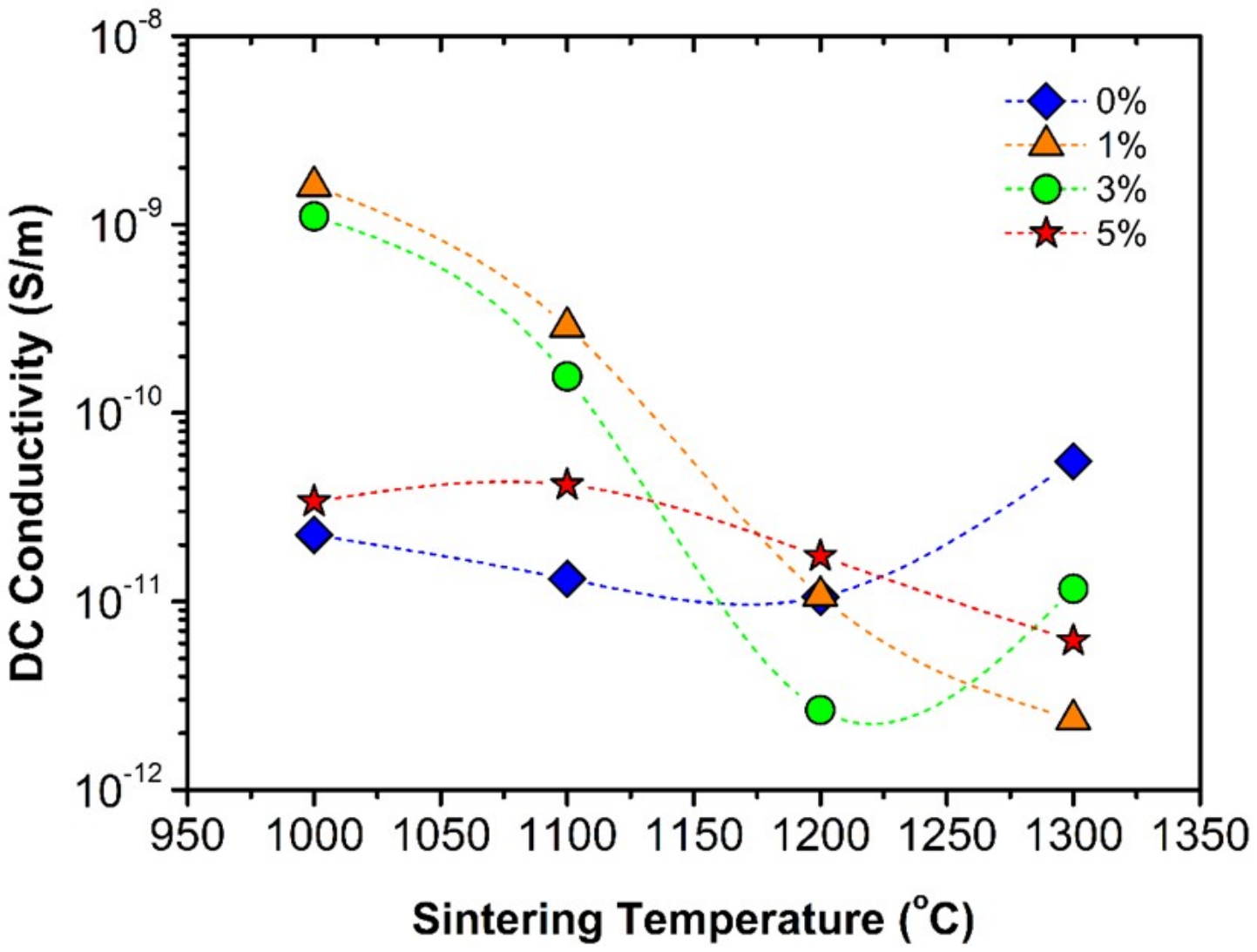

Figure S14. Variation of the DC conductivity of HAp/TiO_2 versus the sintering temperature for 1–5 wt.% TiO_2.

The AC conductivity increases gradually with frequency in the range of 10–100 kHz, exhibiting an approximately logarithmic dependence in the investigated frequency range. This behavior is characteristic of hopping-type conduction in defect-controlled ceramic systems and can be described by Jonscher's universal power law:

$$\sigma_{ac}(\omega) \equiv \sigma - \sigma_{dc} = a\omega^n$$

where ω is the angular frequency, a is a temperature-dependent constant, and n is the frequency exponent associated with charge-carrier dynamics. Parameters a and n are tabulated in Table S3 for the temperatures of 1000-1300 °C, and 0 and 5 wt.% TiO_2.

In the conventional Jonscher universal dielectric response,

$$0 < n < 1.0.$$

Therefore, the values between 0.82 and 0.97 are physically acceptable and support dispersive conductivity associated with localized hopping or polarization-assisted motion.

The values $n = 1.03$ and especially $n = 1.09$ are slightly greater than unity. They should not automatically be considered erroneous, but they are outside the conventional sublinear Jonscher regime. Such values may arise from electrode or interfacial polarization, overlap of more than one relaxation process, uncertainty in the nonlinear fitting, or a superlinear power-law contribution at higher frequencies.

Table S4. Parameters *a* and *n* of Jonscher's power law, $\sigma_{ac}(\omega) = a\omega^n$ for the sintering temperatures of 1000-1300 °C, and 0 and 5 wt.% TiO_2.

**0 wt%**	***a* (S/m)**	***n***	**R^2**
**1000**	7.49E-11	0.97	0.95
**1100**	3.48E-11	1.03	0.93
**1200**	2.80E-10	0.87	0.97
**1300**	2.21E-10	0.85	0.96
**5 wt%**			
**1000**	2.23E-11	1.09	0.94
**1100**	5.46E-10	0.82	0.95
**1200**	2.19E-10	0.84	0.95
**1300**	2.60E-10	0.85	0.94
